# A specific box switches the cell fate determining activity of XOTX2 and XOTX5b in the *Xenopus *retina

**DOI:** 10.1186/1749-8104-2-12

**Published:** 2007-06-27

**Authors:** Marco Onorati, Federico Cremisi, Yang Liu, Rong-Qiao He, Giuseppina Barsacchi, Robert Vignali

**Affiliations:** 1 Dipartimento di Biologia, Unità di Biologia Cellulare e dello Sviluppo, Università di Pisa, Via G. Carducci 13, 56010 Ghezzano (Pisa), Italy; 2 Scuola Normale Superiore, Piazza dei Cavalieri 7, 56100 Pisa, Italy; 3 State Key Lab of Brain and Cognitive Sciences, Institute of Biophysics, Chinese Academy of Sciences, Da Tun Road, Chao Yang District, Beijing 100101, China RP; 4 Dana-Farber Cancer Institute, Jimmy Fund Way, Boston, MA 02115, USA; 5 AMBISEN Center, High Technology Center for the Study of the Environmental Damage of the Endocrine and Nervous System, Università di Pisa, Pisa, Italy

## Abstract

**Background:**

*Otx *genes, orthologues of the *Drosophila orthodenticle *gene (*otd*), play crucial roles in vertebrate brain development. In the *Xenopus *eye, *Xotx2 *and *Xotx5b *promote bipolar and photoreceptor cell fates, respectively. The molecular basis of their differential action is not completely understood, though the carboxyl termini of the two proteins seem to be crucial. To define the molecular domains that make the action of these proteins so different, and to determine whether their retinal abilities are shared by *Drosophila *OTD, we performed an *in vivo *molecular dissection of their activity by transfecting retinal progenitors with several wild-type, deletion and chimeric constructs of *Xotx2*, *Xotx5b *and *otd*.

**Results:**

We identified a small 8–10 amino acid divergent region, directly downstream of the homeodomain, that is crucial for the respective activities of XOTX2 and XOTX5b. In lipofection experiments, the exchange of this 'specificity box' completely switches the retinal activity of XOTX5b into that of XOTX2 and *vice versa*. Moreover, the insertion of this box into *Drosophila *OTD, which has no effect on retinal cell fate, endows it with the specific activity of either XOTX protein. Significantly, in cell transfection experiments, the diverse ability of XOTX2 and XOTX5b to synergize with NRL, a cofactor essential for vertebrate rod development, to transactivate the rhodopsin promoter is also switched depending on the box. We also show by GST-pull down that XOTX2 and XOTX5b differentially interact with NRL, though this property is not strictly dependent on the box.

**Conclusion:**

Our data provide molecular evidence on how closely related homeodomain gene products can differentiate their functions to regulate distinct cell fates. A small 'specificity box' is both necessary and sufficient to confer on XOTX2 and XOTX5b their distinct activities in the developing frog retina and to convert the neutral orthologous OTD protein of *Drosophila *into a positive and specific XOTX-like retinal regulator. Relatively little is known of what gives developmental specificity to homeodomain regulators. We propose that this box is a major domain of XOTX proteins that provides them with the appropriate developmental specificity in retinal histogenesis.

## Background

The vertebrate neural retina is made up of six main types of neurons (cone, rod, horizontal, bipolar, amacrine and retinal ganglion cells) plus the Müller glia cells. All these cell types are generated from a pool of multipotent retinal progenitor cells (RPCs) in a precise time schedule that is largely conserved among different vertebrates [1-4]. The molecular mechanisms driving the RPCs toward specific cell fates are under intense scrutiny and several transcription factors play a crucial role in this process. Basic helix-loop-helix (bHLH) and homeodomain factors are known to regulate the competence state of RPCs (the ability to generate one or more types of neurons), or to more directly address RPCs toward specific cell fates [5-19].

Among the homeobox genes involved in retinal cell fate regulation are the *Otx *genes *Otx2 *and *Crx/Otx5*. These genes are related to the *orthodenticle (otd*) gene of *Drosophila*, required for normal anterior development of the fly [20,21]. Two *otx *genes, *Otx1 *and *Otx2*, were initially isolated in mouse [22] and shown to be essential for correct development of the rostral brain; the *Otx2*^-/- ^phenotype is especially severe, leading to complete lack of anterior structures [23-26]. This phenotype can be rescued by the *Drosophila otd *gene [27,28]. Conversely, the effects of *otd *mutation in *Drosophila *are rescued by either human *OTX1 *or *OTX2 *[29,30]. Finally, *Otx1 *and *Otx2 *seem interchangeable with respect to many aspects of mouse anterior development [31]. These data suggested an extensive functional conservation of the OTX/OTD class of proteins.

*Crx *is an *otx*-like gene important for the differentiation and maintenance of photoreceptors [32,33]. The CRX protein is able to bind and activate photoreceptor specific genes, such as those encoding interphotoreceptor retinoid-binding protein (*IRBP*), β-phosphodiesterase, arrestin and opsin [33,34]. CRX biological activity greatly depends on molecular interactions with partners such as NRL, an essential cofactor for vertebrate rod development [35]. These interactions were shown *in vitro *[36], and in transfection assays that demonstrate a synergy of the two factors in activating photoreceptor gene promoters [37]. Mutations in *CRX *are associated with diverse human eye diseases [33,38-42], and some affect CRX-NRL interaction and/or CRX transactivating ability [36,43]. In mouse, *Crx *function seems essential for terminal differentiation: in *Crx*^-/- ^mice, photoreceptors do not develop their outer segments and display perturbed synaptogenesis [44,45]. However, though defective, photoreceptors do initially develop in *Crx*^-/- ^mice, suggesting that their commitment relies on other players. In particular, results of conditional *Otx2 *loss-of-function in the mouse retina suggest that *Otx2 *controls photoreceptor initial specification and activates *Crx *expression in committed precursors [46].

A different picture is present in other vertebrates. In *Xenopus*, *Xotx2 *and *Xotx5b *(the homolog of *Crx*) are expressed in different patterns during retinal histogenesis: transcription of both genes starts at tailbud stage in a diffused fashion throughout the retina, but then their expression is progressively restricted and, in the mature retina, *Xotx2 *mRNA is found only in bipolar cells, while *Xotx5b *is transcribed in both photoreceptors and a subset of bipolar cells [12]. Even more dramatic is the difference in the protein expression pattern: XOTX2 protein is detected only in bipolar cells, while XOTX5b is produced only in photoreceptors, due to precise translational control through the 3' untranslated regions (UTRs) of their mRNAs [47]. Consistent with the pattern of protein distribution, lipofection of RPCs with constitutively expressed *Xotx2 *and *Xotx5b *cDNAs lacking the 3' UTR showed dramatically different effects, with *Xotx2 *driving cells toward bipolar cell fate, and *Xotx5b *toward photoreceptor cell fate [12,17,47]. Interestingly, domain-swapping experiments showed that the specific effect of either protein relies on its carboxyl terminus [12]. Because of the substantial similarity of the two proteins (XOTX2 and XOTX5b are 75% identical overall; 96% identical in the homeodomain) and of the functional conservation between *Otx/otd *genes in regulating early developmental events, we asked the following questions: which part of the XOTX2 or XOTX5b protein is crucial for their specific activities driving RPCs toward bipolar or photoreceptor cell type, respectively? Is *Drosophila *OTD able to drive RPCs toward any specific cell fate in the *Xenopus *retina?

To answer these questions, we performed an *in vivo *molecular dissection of the activity of several wild-type and mutant *Xotx2 *and *Xotx5b *constructs on retinal cell specification. We thus identified, directly downstream of the homeodomain, a small 8–10 amino acid divergent region that is both necessary and sufficient for XOTX2 and XOTX5b specific activities on cell fate; this region works as a 'specificity box' that switches the retinal activity of XOTX5b into that of XOTX2 and *vice versa*. Significantly, the insertion of this box into *Drosophila *OTD, which has no cell fate effect in the frog retina, endows it with the retinal activity of either XOTX2 or XOTX5b. We also found that the greater ability of XOTX5b, compared to XOTX2, to synergize with *Xenopus *NRL (XNRL) to activate the rhodopsin promoter is also switched depending on this box. We next investigated whether these results may be due to differential interactions of XOTX/OTD proteins with XNRL: we found that XOTX2 and XOTX5b differentially interact with XNRL, but also that OTD and mutant XOTX/OTD proteins are able to bind XNRL, suggesting that this domain is not essential for XOTX interactions with XNRL, though it may modulate XOTX/OTD interactions with XNRL and their overall specific actions. Our data provide *in vivo *and *in vitro *molecular evidence on how closely related homeodomain factors differentiate their functions to regulate distinct cell fates.

## Results

### A small 8–10 amino acid region confers specific activities on XOTX2 and XOTX5b in the *Xenopus *retina

**Figure 1:**
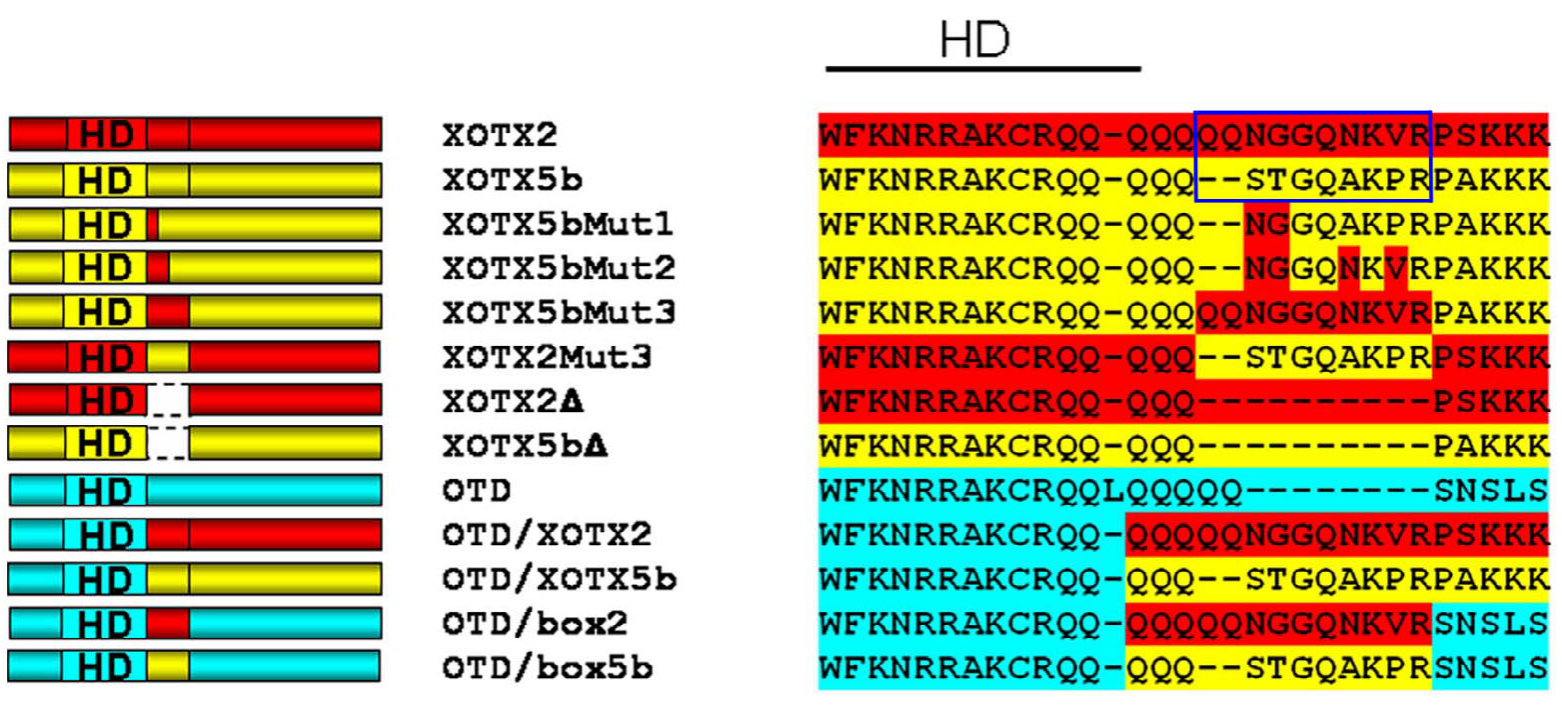
Constructs used in this study. On the left are schematics of the different constructs; on the right are their sequences in the final region of the homeodomain (HD) and directly downstream of it, with different colors shading the parental sequences of XOTX2 (red), XOTX5b (yellow) and OTD (blue). Lines are introduced for sequence alignment. The divergent region responsible for the different retinal activities of XOTX2 and XOTX5b (RS box) is shown in the blue box.

Previous lipofection experiments showed that two chimeric constructs, *Xotx2/engR *and *Xotx5b/engR*, in which most of the transactivation domain of either XOTX2 or XOTX5b was replaced with the repressor domain of the *Drosophila *Engrailed protein, had specific effects on either bipolar cells or photoreceptor cells, respectively, in this case leading to a decrease, rather than an increase, in their frequency [12]. This observation suggested that these XOTX/engR chimeric proteins might retain a region of XOTX2 or XOTX5b crucial for their specific activities, and focused our attention on the only part of their carboxyl termini that was included in the *Xotx2/engR *and *Xotx5b/engR *constructs. This region spans amino acids 100–109 of XOTX2 and amino acids 100–107 of XOTX5b, where the two proteins differ in six residues (Figure 1).

**Figure 2:**
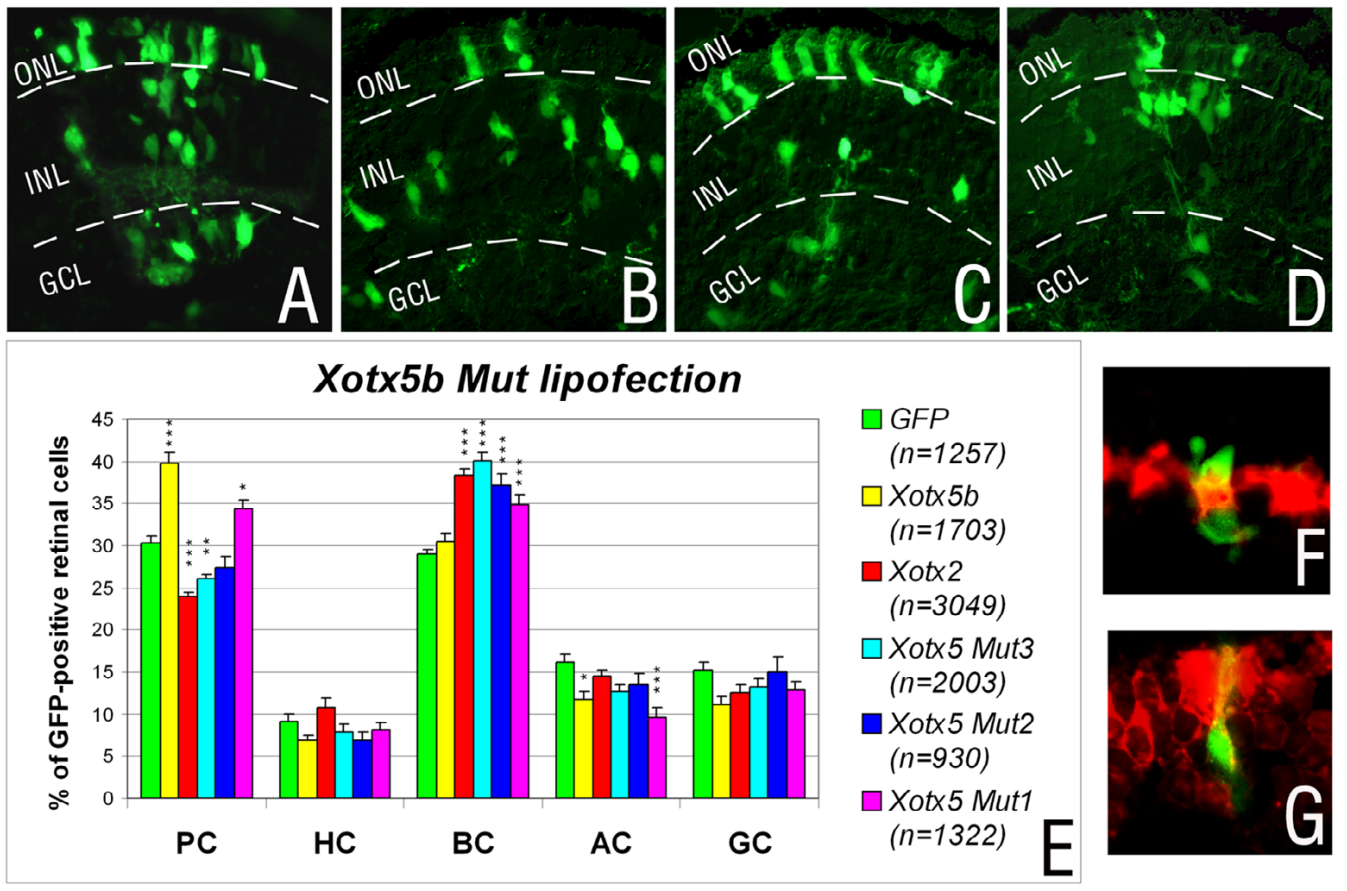
Results of *in vivo *lipofection of RPCs with wild-type *Xotx2 *and *Xotx5b*, and mutant *Xotx5b *constructs. **(a-d) **Sample sections are shown for control retinae lipofected with *GFP*+vector DNA alone (a), *GFP+Xotx2 *(b), *GFP+Xotx5b *(c) or *GFP+Xotx5bMut3 *(d); GCL, ganglion cell layer; INL, inner nuclear layer; ONL, outer nuclear layer. **(e) **Overall distribution of retinal cell types in clones lipofected with the different constructs; PC, photoreceptor cells; HC, horizontal cells; BC, bipolar cells; AC, amacrine cells; GC, ganglion cells. The proportion of each cell type is represented as an average. Error bars indicate the standard error of the mean. The experiment was repeated at least three times for all constructs. Counted cells are indicated in the histogram (*n*), from 15 retinae for *GFP*, 15 retinae for *Xotx5b*, 18 retinae for *Xotx2*, 16 retinae for *Xotx5bMut3*, 10 retinae for *Xotx5bMut2*, and 13 retinae for *Xotx5bMut1*. Asterisks represent significant differences between *Xotx *constructs and *GFP*, as calculated by ANOVA analysis using the Tukey-Kramer post-test (*p < 0.05, **p < 0.01, ***p < 0.001). **(f, g) ***In situ *hybridization analyses showing examples of GFP-positive (green), *Xotx5*-lipofected photoreceptor cell positive for *IRBP *probe (Fast Red detection) (f), and a *Xotx5bMut3*-lipofected bipolar cell expressing *Xvsx1 *(Fast Red detection) (g).

To test if these residues are crucial for the respective activities of the two factors, we changed the XOTX5b amino acid sequence in this region into the corresponding one of XOTX2, and *vice versa*. We first generated three sequential constructs encoding mutant forms of XOTX5b, in which two (construct *Xotx5bMut1*), four (*Xotx5bMut2*), or six (*Xotx5bMut3*) amino acid residues of the relevant region were changed to those of XOTX2, thereby switching this region of XOTX5b into that of XOTX2 (Figure 1). These constructs were lipofected in RPCs and their activities compared to those of wild-type *Xotx2 *and *Xotx5b *(Additional files 1 and 2). As expected [12], lipofections with wild-type *Xotx5b *or *Xotx2 *constructs promoted photoreceptor or bipolar cell fate, respectively (Figure 2a–c, e). On the other hand, unlike wild-type *Xotx5b*, the *Xotx5bMut3 *construct yielded the same effect as wild-type *Xotx2*, increasing bipolar cell and decreasing photoreceptor frequency (Figure 2d, e). Interestingly, lipofections of RPCs with the *Xotx5bMut2 *construct (four amino acid change) increased bipolar cells, but did not decrease photoreceptors; even more interestingly, the *Xotx5bMut1 *construct (two amino acid change) increased bipolar as well as photoreceptor cells, therefore showing the joint effects of both parental proteins (Figure 2e). Molecular markers confirmed the identity of cells lipofected with these different constructs: in particular, cells lipofected with *Xotx5b *and scored as photoreceptors expressed *IRBP*, and were thus *bona fide *photoreceptors and not displaced cells with a different identity (Figure 2f); on the other hand, cells transfected with *Xotx5bMut3 *and scored as bipolar cells expressed the bipolar marker *Xvsx1 *[48] (Figure 2g). Other markers were also used in this study to confirm our diagnoses of different cell types (Additional file 3).

**Figure 3:**
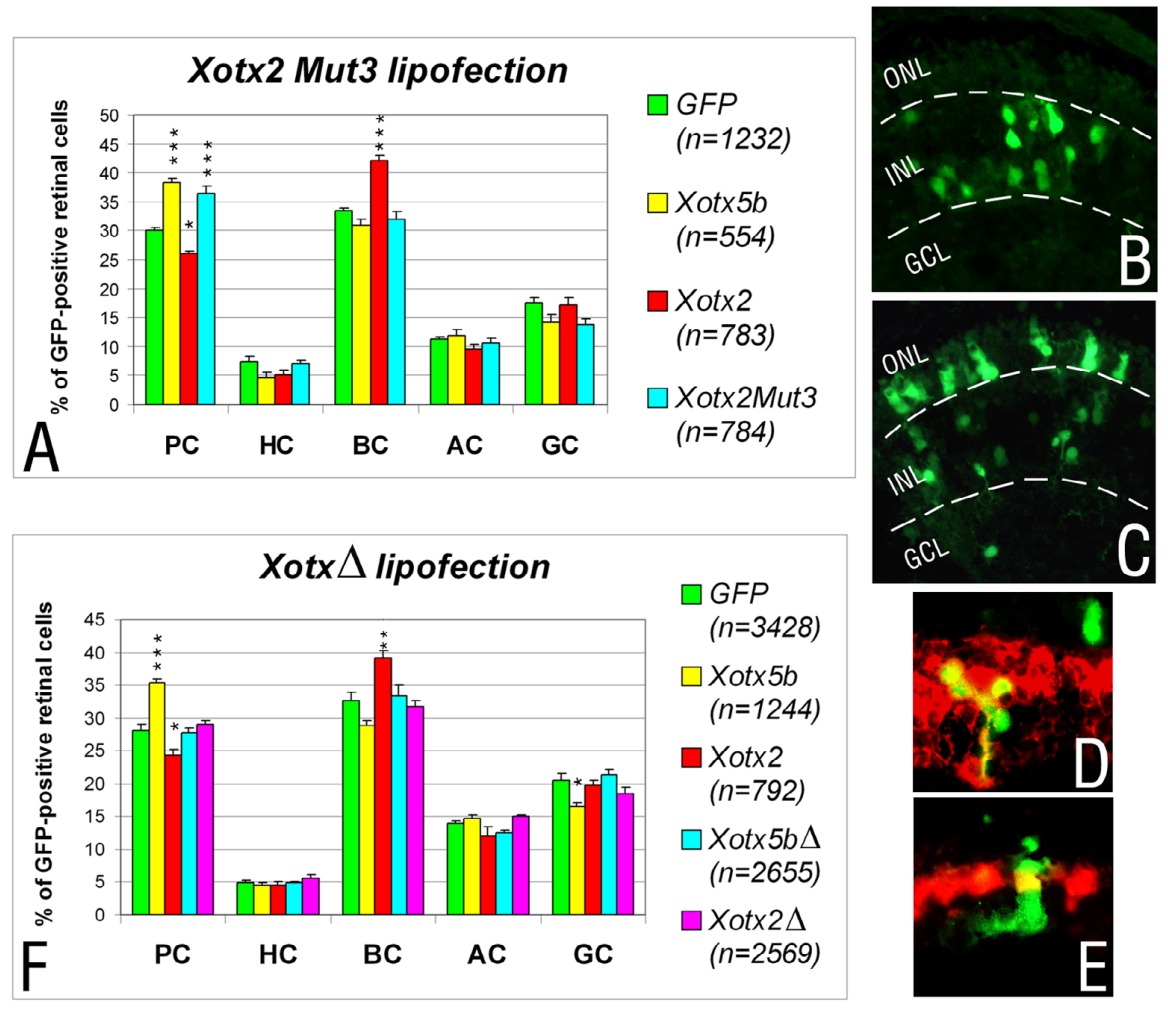
The 'specificity box' downstream of the homeodomain is necessary and sufficient for specific retinal action of XOTX2 and XOTX5b. **(a-c) **Results of *in vivo *lipofection of RPCs with wild-type *Xotx2 *and *Xotx5b*, and mutant *Xotx2Mut3 *constructs, showing the overall distribution of retinal cell types in clones lipofected with the different constructs, as indicated (a); PC, photoreceptor cells; HC, horizontal cells; BC, bipolar cells; AC, amacrine cells; GC, ganglion cells. The proportion of each cell type is represented as average ± standard error of the mean. Counted cells are indicated in the histogram (*n*), from 9 retinae for *GFP*, 10 retinae for *Xotx5b*, 9 retinae for *Xotx2*, and 14 retinae for *Xotx2Mut3*. Asterisks represent significant differences between *Xotx *constructs and *GFP*, as calculated by Tukey-Kramer test (*p < 0.05, **p <0.01, ***p <0.001). Sample sections are shown for retinae co-lipofected with *GFP+Xotx2 *(b) and *GFP+Xotx2Mut3 *(c); GCL, ganglion cell layer; INL, inner nuclear layer; ONL, outer nuclear layer. **(d, e) ***In situ *hybridization analyses showing examples of GFP-positive (green), *Xotx2*-lipofected bipolar cell expressing *Xvsx1 *(Fast Red detection) (d), and *Xotx2Mut3*-lipofected photoreceptor cell positive for *IRBP *probe (Fast Red detection) (e). **(f) **The RS box is required for the biological action of either XOTX2 or XOTX5b proteins: the histogram reports the overall distribution of retinal cell types in clones lipofected with the different constructs, as indicated. Counted cells are indicated in the histogram (*n*), from 11 retinae for *GFP*, 9 retinae for *Xotx5b*, 6 retinae for *Xotx2*, 8 retinae for *Xotx2Δ*, and 7 retinae for *Xotx5Δ*.

We also switched *Xotx2 *into *Xotx5b*. For this, we generated a mutant *Xotx2 *construct (*Xotx2Mut3*) in which the crucial region of XOTX2 was converted to that of XOTX5b (Figure 1). The activity of *Xotx2Mut3 *in lipofections was essentially identical to that of *Xotx5b*: instead of promoting bipolar cell fate like *Xotx2*, the mutant *Xotx2Mut3 *construct promoted photoreceptor fate (Figure 3a–e; Additional files 4 and 5). The identity of photoreceptors generated by RPCs lipofected with *Xotx2Mut3 *was confirmed by testing the expression of the *IRBP *marker (Figure 3e). We conclude that this small region works as a 'retinal specificity (RS) box' that is sufficient to confer on the XOTX2 and XOTX5b proteins their ability to drive RPCs toward specific fates.

We next asked whether the RS box is required for XOTX protein activity in retinal cell fate specification, or whether XOTX proteins still possess a retinal 'default' activity without it. To test this, we generated deletion constructs (*Xotx2Δ *and *Xotx5bΔ*) by removing the RS box (Figure 1), and compared their activities to that of wild-type constructs. Deletion of the RS box completely abrogates any biological effect of either XOTX2 or XOTX5b, showing that this small region is required for their activity in the frog retina (Figure 3f; Additional files 6 and 7).

### The RS box confers specific retinal activities on *Drosophila *OTD

**Figure 4:**
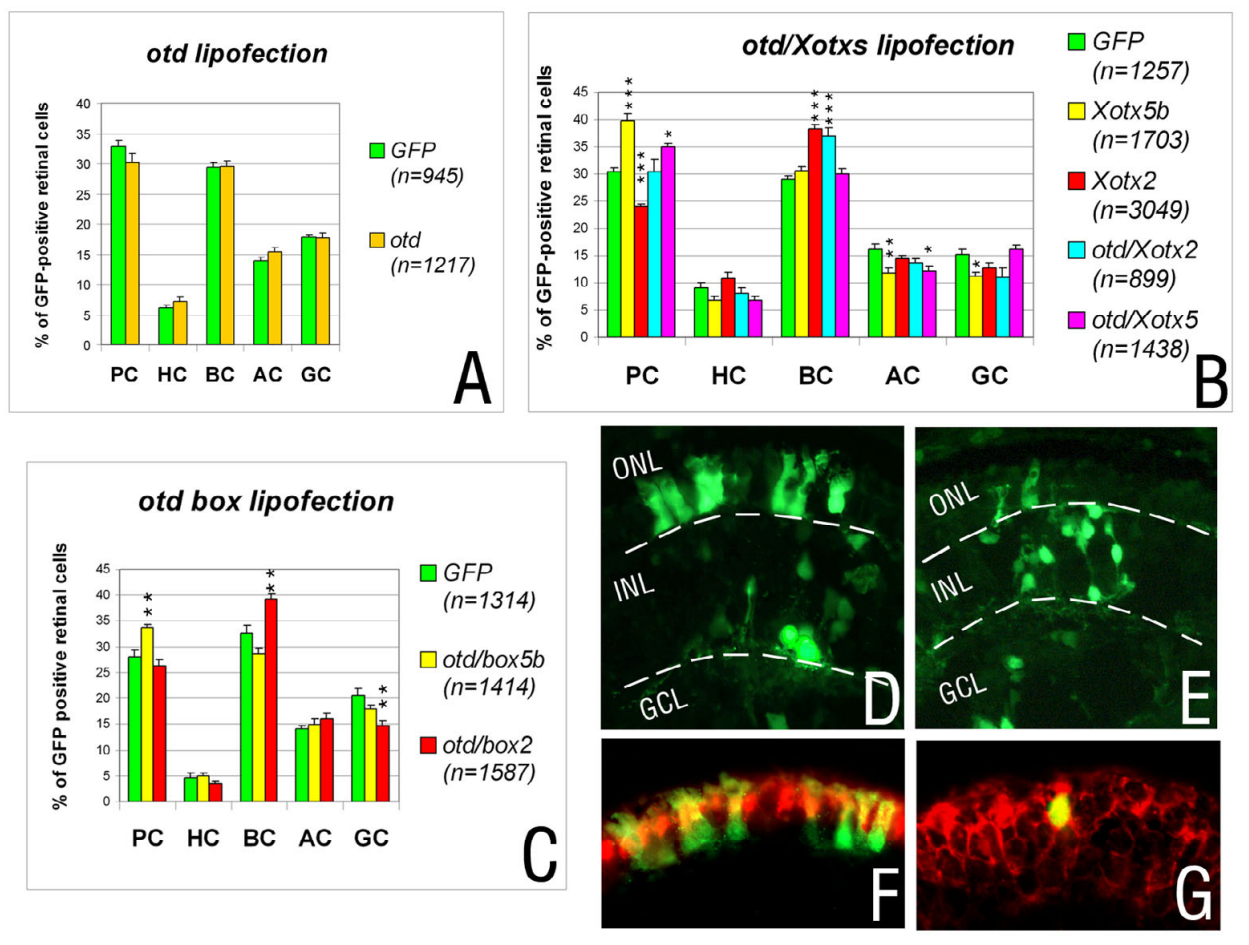
The RS box is sufficient to confer a specific retinal action on *Drosophila *OTD protein. **(a) **Results of lipofection of RPCs with *GFP *alone or with *GFP+otd*; counted cells were as indicated in the histogram (*n*), from 17 retinae for *GFP*, and 11 retinae for *otd*; PC, photoreceptor cells; HC, horizontal cells; BC, bipolar cells; AC, amacrine cells; GC, ganglion cells. **(b) **Results of lipofection of RPCs with *GFP *alone, with *GFP+Xotx *wild-type constructs or *GFP+otd/Xotx *chimeric constructs, as indicated; counted cells are indicated in the histogram (*n*), from 15 retinae for *GFP*, 18 retinae for *Xotx2*, 15 retinae for *Xotx5b*, 9 retinae for *otd/Xotx2*, and 11 retinae for *otd/Xotx5b*. **(c) **Results of lipofection of RPCs with *GFP *alone, or with *GFP+otd/box2/5b *chimeric constructs, as indicated. Counted cells are indicated in the histogram (*n*), from six retinae for *GFP*, nine retinae for *otd/box2*, and nine retinae for *otd/box5b*. The proportion of each cell type (a-c) is represented as average ± standard error of the mean. **(d, e) **Lipofected retinae with *otd/box5b *are enriched in photoreceptors (d), those with *otd/box2 *are enriched in bipolar cells (e); GCL, ganglion cell layer; INL, inner nuclear layer; ONL, outer nuclear layer. **(f) **An example of *GFP+otd/box5b*-lipofected photoreceptor cell positive for *IRBP *probe after *in situ *hybridization (Fast Red detection). **(g) **A *GFP+otd/box2*-lipofected bipolar cell expressing *Xvsx1 *is shown following *in situ *hybridization (Fast Red detection).

Because of the extensive functional conservation of OTX/OTD proteins in early development of the anterior region of fly and mouse embryos, we asked whether *Drosophila *OTD was able to direct RPCs to any specific cell fate in the *Xenopus *retina. In lipofections, no difference was observed in the frequency of the different retinal cell types between *otd *lipofected and control clones (Figure 4a; Additional file 1). The carboxyl terminus of OTD is quite divergent compared to XOTX2 or XOTX5b (it shares only 11.4% identity with XOTX2 and only 8% with XOTX5b); instead, in the same region, XOTX2 and XOTX5b are 75% identical, suggesting that a possible reason for the lack of OTD activity could be due to such a strong divergence, and possibly to absence of the box in the OTD protein (Figure 1). To test this, we first replaced the entire OTD region carboxy-terminal to the homeodomain with that of either XOTX2 or XOTX5b, and compared the activities of chimeric OTD/XOTX2 and OTD/XOTX5b (Figure 1) with those of XOTX2 and XOTX5b. Significantly, OTD/XOTX2 drives RPCs toward the bipolar cell fate, while OTD/XOTX5b increased photoreceptors (Figure 4b). However, the decrease in photoreceptor cell frequency produced by wild-type *Xotx2 *was not observed with the *otd/Xotx2 *chimeric construct (Figure 4b; Additional files 1 and 8).

We then tested whether the RS box is sufficient to confer the biological activity of either XOTX2 or XOTX5b on *Drosophila *OTD. To do this, we generated chimeric *otd/box2 *and *otd/box5b *contructs in which the XOTX2 or XOTX5b RS box, respectively, was inserted immediately downstream of the OTD homeodomain (Figure 1), and transfected them into *Xenopus *RPCs. Though the carboxyl terminus of OTD is strongly divergent from those of both XOTX proteins, the specificity box enabled OTD/box2 or OTD/box5b proteins to drive RPCs toward bipolar or photoreceptor fates, respectively (Figure 4c–g). Similar to OTD/XOTX2, OTD/box2 did not lead to a significant reduction in photoreceptor cells (Figure 4c). No other effect was observed on the frequencies of the remaining cell types, with the exception of a decrease of ganglion cells with OTD/box2 (Figure 4c; Additional files 9 and 10).

**Figure 5:**
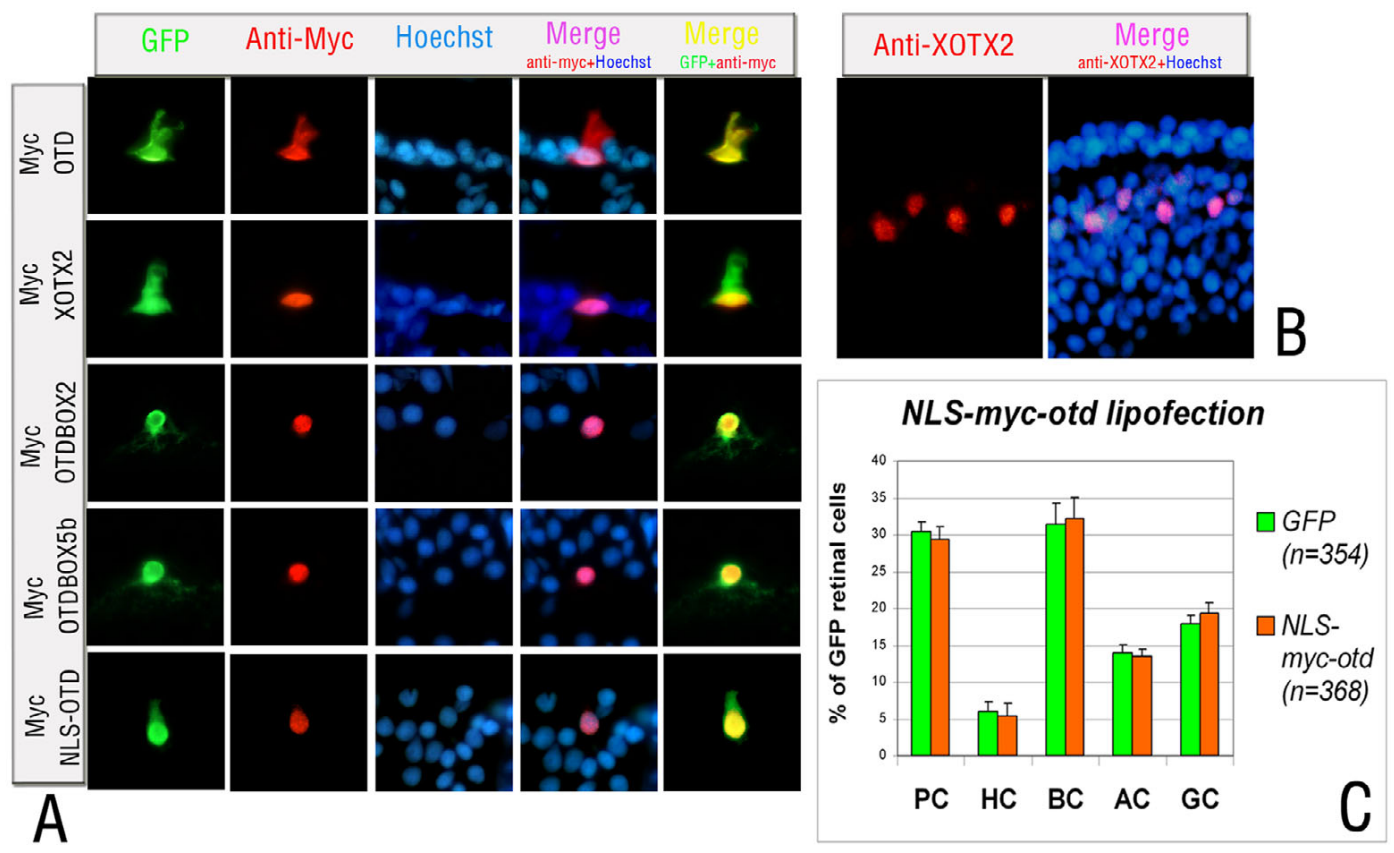
The effect of the specificity box upon commitment of RPCs is not simply due to possible effects on nuclear localization. **(a) **The nuclear/cytoplasmic distribution of MYC-XOTX2 and MYC-OTD (as indicated at the left) is shown in lipofected retinal cells, and is compared to cytoplasmic GFP fluorescence; while MYC-XOTX2 shows an exclusively nuclear localization, MYC-OTD is partly cytoplasmic; on the contrary, MYC-OTD/box2 and MYC-OTD/box5b are targeted to the nucleus; a NLS-MYC-OTD fusion protein is forced into the nucleus (bottom row). **(b) **Endogenous XOTX2 protein distribution is detected by a specific antibody (red fluorescence) in the *Xenopus *retinal nuclei. **(c) **Although it forces OTD to the nucleus, a *NLS-Myc-otd *fusion construct does not have any effect on cell fate of RPCs. Counted cells were as indicated in the histogram (*n*), from six retinae for *GFP*, and four retinae for *NLS-Myc-otd*. PC, photoreceptor cells; HC, horizontal cells; BC, bipolar cells; AC, amacrine cells; GC, ganglion cells. Nuclei are counterstained with Hoechst.

Previous work on CRX showed evidence of an additional nuclear localization signal (NLS) at the carboxyl terminus of the homeodomain. Several CRX mutations are associated with human retinal pathologies and have an effect on CRX nuclear localization [49]; one such mutation, leading to CRX mislocalization, replaces Arg98 with leucine. Because a leucine residue is present in *Drosophila *OTD at this position (Figure 1), we asked whether the inactivity of OTD in retinal specification was due to insufficient translocation to the nucleus, rather than to the absence of the RS box. To test this, we compared the distributions of MYC-XOTX2, MYC-XOTX5b, MYC-OTD, MYC-OTD/box2 and MYC-OTD/box5b proteins in lipofected retinal cells, detected with an anti-MYC antibody, with those of endogenous XOTX2 and XOTX5b, detected by specific antibodies [47]. Endogenous XOTX2 and XOTX5b were found only in the nuclei of bipolar and photoreceptor cells, respectively, but not in the cytoplasmic compartment of these or other retinal cells (Figure 5b; data not shown for XOTX5b). A nuclear distribution was found for MYC-XOTX2 and MYC-XOTX5b in all lipofected cells (Figure 5a; data nor shown for MYC-XOTX5b). Interestingly, while MYC-OTD was present in both nuclear and cytoplasmic compartments of lipofected cells, MYC-OTD/box2 and MYC-OTD/box5b had a clear nuclear localization (Figure 5a).

While these results suggest that the RS box might be important for correct nuclear targeting of XOTX proteins, the possibility remained that potential OTD effects on cell fate did not occur due to insufficient translocation of it to the nucleus. To rule out this possibility, we lipofected a *NLS-myc-otd *construct (containing a nuclear localization signal) to force OTD to the nucleus, and evaluated its ability to drive RPCs to specific cell fates. As expected, the NLS-MYC-OTD protein was correctly localized to the nucleus (Figure 5a), but it did not significantly affect cell fate (Figure 5c; Additional file 11). Therefore, efficient translocation to the nuclei of retinal cells did not provide OTD with the ability to drive frog RPCs toward a precise neuronal fate; instead, OTD gained this ability when its homeodomain was directly followed by a RS box of the XOTX2 or XOTX5b type.

### XOTX2 and XOTX5b differentially synergize with XNRL to transactivate the rhodopsin promoter

**Figure 6:**
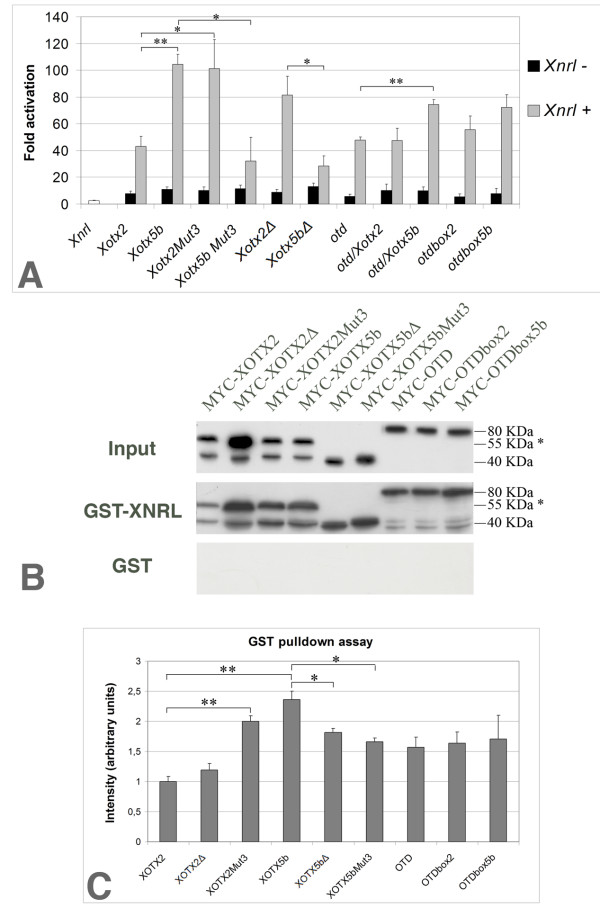
XOTX2 and XOTX5b differentially synergize with XNRL to activate the rhodopsin promoter and differentially interact *in vitro *with XNRL. **(a) **Results of rhodopsin promoter cell transfection assays with several *Xotx/otd *constructs (with or without *Xnrl*). Error bars indicate the standard error of the mean. **(b) **GST-pull down assays compare the interaction of MYC-XOTX/OTD fusion proteins to GST-XNRL or GST alone. The band indicated by an asterisk corresponds to a higher molecular weight (55 kDa) than the one expected for XOTX proteins (41 kDa) and may result from post-translational modification; this needs further investigation. **(c) **Results of two of these experiments, analyzed by Image J, were statistically processed; columns show the ratio of the retained MYC-tagged proteins relative to their respective input, normalized with respect to the MYC-XOTX2 retained/input ratio. Error bars indicate the standard deviation. The *p*-value was calculated by bilateral Student's *t*-test. Asterisks in histograms show statistically significant differences only for more relevant comparisons (*p < 0.05, **p <0.01). (see Additional files 12 and 13 for the full data set).

Previous work showed that CRX/XOTX5b interacts with NRL to activate the rhodopsin promoter [36,50], and that a lower level of activation is obtained when *Otx2 *and *Nrl *are co-transfected in cultured cells [37]. We therefore asked whether the *Xotx *mutant constructs also switched their activity in similar transfection assays. We thus monitored the ability of XOTX2 and XOTX5b (alone or together with XNRL) to activate a *Xenopus *rhodopsin promoter (XOP) driving green fluorescent protein (GFP) expression in HEK 293T cells, and compared it with the activities of XOTX5bMut3, XOTX2Mut3, OTD, OTD/XOTX2, OTD/XOTX5b, OTD/box2, OTD/box5b, XOTX2Δ and XOTX5bΔ. When each of these constructs was co-transfected with the XOP-GFP reporter [50] in the absence of XNRL, scarce activation of the reporter gene was detected (5- to 13-fold activation compared to the ground level given by transfection of XOP-GFP alone); the same held true for transfection of *Xnrl *alone (Figure 6a; Additional file 12). A significant difference (*p *< 0.01, Student's *t*-test) was observed between *Xotx2+Xnrl *and *Xotx5b+Xnrl *transfections, which elicited activation of the co-transfected reporter 43- and 105-fold over the ground level, respectively; these results are consistent with those of Peng and Chen [37].

More significantly, *Xotx2Mut3+Xnrl *transfection gave similar results to *Xotx5b+Xnrl *(101-fold reporter activation; *p *= 0.035 for *Xotx2Mut3+Xnrl *versus *Xotx2+Xnrl*; *p *= 0.89 for *Xotx2Mut3+Xnrl *versus *Xotx5b+Xnrl*), while *Xotx5bMut3+Xnrl *(32-fold activation) gave similar results to *Xotx2+Xnrl *(*Xotx5bMut3+Xnrl *versus *Xotx2+Xnrl*, *p *= 0.55; *Xotx5bMut3+Xnrl *versus *Xotx5b+Xnrl*, *p *= 0.02). Therefore, exchanging the RS box in XOTX2 or XOTX5b leads to a switch in their ability to transactivate, together with XNRL, one of the key photoreceptor specific genes. In addition, *otd*, *otd/Xotx2 *or *otd/box2*, when combined with *Xnrl*, all gave results similar to *Xotx2 *(48-, 47- and 56-fold activation, respectively); a slightly stronger effect was observed with *otd/Xotx5b+Xnrl *(*otd/Xotx5b+Xnrl *versus *otd+Xnrl*, *p *= 0.003) and *otd/box5+Xnrl *(74- and 72-fold activation, respectively). Surprisingly, a rather strong effect was obtained with *Xotx2Δ+Xnrl *(82-fold activation; *Xotx2Δ+Xnrl *versus *Xotx2+Xnrl*, *p *= 0.047; *Xotx2Δ+Xnrl *versus *Xotx5b+Xnrl*, *p *= 0.22; *Xotx2Δ+Xnrl *versus *Xotx5bΔ+Xnrl*, *p *= 0.029), but not with *Xotx5bΔ+Xnrl *(28-fold activation; *p *= 0.014 versus *Xotx5b+Xnrl*). Significant differences were also found between *Xotx5b+Xnrl *and *otd+Xnrl *(*p *= 0.002).

### *In vitro *interactions of XOTX/OTD proteins with XNRL

One possible way to explain the different activities of XOTX2 and XOTX5b is that the two proteins differentially interact with other key molecular players involved in retinal differentiation, such as XNRL itself. Full-length CRX, or truncated CRX forms containing the homeodomain, the Q-rich region and the basic region altogether, were shown to strongly interact with NRL, whereas the CRX homeodomain alone showed much lower interaction [36].

Because the RS box spans from the Q-rich region to part of the basic region [51], we decided to test whether XOTX2 and XOTX5b show differential interaction with XNRL. We therefore prepared a GST-XNRL fusion construct and purified the corresponding protein; this was used in GST-pull down assays against affinity purified full-length MYC-XOTX2, MYC-XOTX2Δ, MYC-XOTX2Mut3, MYC-XOTX5b, MYC-XOTX5bΔ, MYC-XOTX5bMut3, MYC-OTD, MYC-OTD/box2, and MYC-OTD/box5b. The results of these experiments are shown in Figure 6b; the pull-down results were scanned and analyzed by Image J [52], and the resulting data were plotted (Figure 6c; Additional file 13). We found that MYC-XOTX5b interacts with GST-XNRL about 2.4-fold more compared to MYC-XOTX2 (*p *< 0.01, Student's *t*-test); significant differences were also observed compared to MYC-XOTX2Δ, MYC-XOTX5bΔ, MYC-XOTX5bMut3, MYC-OTD and MYC-OTD/box2, but not compared to MYC-XOTX2Mut3, or MYC-OTD/box5b. Therefore, XOTX5b interacts more strongly with XNRL, but other XOTX/OTD proteins also interact *in vitro *with XNRL, even without the box.

## Discussion

We have identified a small, divergent region that confers specific retinal activities to XOTX2 and XOTX5b. This RS box lies directly carboxy-terminal to the homeodomain, extending for 8–10 amino acids from the the poly-Q tail to embrace part of the basic region as identified in CRX [51]. Remarkably, this divergent region is necessary and sufficient to confer on these XOTX proteins their specific cell fate specification activity in the frog retina. First, deletion of the box completely abrogated any cell fate activity of both XOTX2 and XOTX5b. Furthermore, exchanging the sequence of the XOTX5b RS box into that of the XOTX2 box (construct *Xotx5bMut3*) and *vice versa *(construct *Xotx2Mut3*) completely switched the biological activities of the two proteins. Two other *Xotx5b *mutant constructs showed interesting intermediate effects: *Xotx5bMut2 *pushed RPCs toward a bipolar cell fate (like *Xotx2*), but had no significant effect on decreasing photoreceptor cell frequency (unlike *Xotx2*); *Xotx5bMut1 *showed the activities of both *Xotx2 *and *Xotx5*, as it was able to increase both bipolar and photoreceptor cells (though in the latter case with significantly lower efficiency than *Xotx5b*). These data show that the first two changes in the XOTX5b amino acid sequence (S100N, T101G) are sufficient to endow XOTX5bMut1 with a great part of the XOTX2 ability to promote bipolar fate; in fact, there was no statistical difference between *Xotx2 *and *Xotx5bMut1 *(*p *> 0.05; or *Xotx5bMut2 *or *Xotx5bMut3*, *p *> 0.05) in their efficiency to promote bipolar cells. This suggests that Asn102 and/or Gly103 are particularly important residues for this aspect of XOTX2 action.

Mutant and wild-type *Xotx5b *constructs also showed graded effects on photoreceptor commitment: *Xotx5bMut1 *promoted, rather then repressed, photoreceptors, similar to *Xotx5b*; *Xotx5bMut2 *showed no effect on photoreceptor frequency; and *Xotx5bMut3 *led to significantly fewer photoreceptors compared to GFP controls. In particular, *Xotx5bMut1 *photoreceptor-promoting activity was significantly lower than that of *Xotx5b*; furthermore, constructs *Xotx5bMut1 *and *Xotx5bMut2 *yielded significantly different effects (*p *< 0.001), whereas no significant difference occurred between constructs *Xotx5bMut2 *and *Xotx5bMut3 *(*p *> 0.05; or these two and *Xotx2*, *p *> 0.05). These results suggest that the changing of the first two amino acids may not have completely compromised the photoreceptor promoting activity of XOTX5b, while the next specific mutagenetic changes led to its abrogation and a reversal of its effect. The six residues may, therefore, have additive roles in determining XOTX2 repressive effect on photoreceptor fate.

We show that wild-type OTD does not mimic XOTX2 or XOTX5b in the frog retina. However, replacement of the OTD carboxyl terminus with that of either XOTX protein, or even the simple insertion of either the XOTX2 or XOTX5b specificity box into OTD, provides it with the activity of XOTX2 or XOTX5b. This is remarkable since OTD lacks some of the functional domains important for the transactivating ability of CRX/OTX proteins, such as the OTX tail and the WSP domain [43,51]. Therefore, the RS box is sufficient to promote specific cell fates in a rather more divergent context than that of vertebrate OTX proteins. This is not due to mere effects of the box on cytoplasmic-nuclear trafficking, because forcing OTD to the nucleus using a NLS does not have any effect on RPC fate and because the effect of OTD/box2 and OTD/box5b specifically depends on the type of RS box. Therefore, we suggest that while the carboxy-terminal domain of OTD mimics, to a certain extent, the transactivating activity of the XOTX2 and XOTX5b carboxyl termini, OTD, in the absence of the RS box, fails to properly target the gene sets that address RPCs to their fates.

However, not all XOTX2 or XOTX5b retinal functions depend on the RS box. Unlike XOTX2, OTD/XOTX2 is unable to repress photoreceptors. This is different from the effect shown by the *Xotx5b/Xotx2 *chimeric construct, which retains *Xotx2 *anti-photoreceptor activity [12], suggesting that some features of XOTX retinal activity may also depend on the amino terminus. XOTX5b and XOTX2 amino-terminal regions (excluding the homeodomain) are about 73% identical, while the OTD amino terminus is only about 15% identical to that of XOTX2. It is possible that the amino terminus of XOTX5b may better match the carboxyl terminus of XOTX2 (and *vice versa*) than the amino terminus of OTD, thus allowing the exploitation of the full spectrum of protein activities. Such activity of the amino terminus could be due to possible interactions with other parts of the XOTX protein at either the intramolecular level, for example, to allow proper folding of the protein, or at the intermolecular level, for example, with other XOTX monomers [53] or other molecular partners.

How can the RS box modulate the activity of XOTX2 and XOTX5b proteins? Two possibilities, not mutually exclusive, are that the box refines the DNA binding abilities of XOTX proteins towards different sets of promoters, or that it modulates interactions with other molecular partners. While the overall effects on cell fate of wild-type and mutant XOTX2 and XOTX5b strongly suggest that different sets of genes are indeed activated depending on the type of RS box, we also show that XOTX2 and XOTX5b differentially synergize with XNRL in the regulation of the rhodopsin promoter, and that this ability is switched by the RS box. Some activation is also observed when XNRL is transfected together with OTD/XOTX5b or OTD/box5b (although this is significant only in the case of OTD/XOTX5b). Instead, XOTX5bΔ, OTD, OTD/XOTX2 and OTD/box2 do not appear to synergize strongly with XNRL on the rhodopsin promoter. While these results are quite consistent with the *in vivo *photoreceptor promoting activity of these constructs, we found, unexpectedly, that XOTX2Δ+XNRL also activates the rhodopsin promoter. This result does not seem completely consistent with the lipofection results, where *Xotx2Δ *does not promote photoreceptor fate. However, the *in vivo *cell fate specification activity of XOTX proteins presumably occurs through the activation of entire sets of genes, and may be more complex that what can be measured from their activity on a single promoter. XOTX2Δ may have some intrinsic ability to act on the rhodopsin promoter together with XNRL; this may normally be impaired by the RS box in XOTX2, and becomes unmasked when the box is removed. On the other hand, XOTX5bΔ does not have this intrinsic ability, and XOTX5b specifically requires the RS box to synergize with XNRL. In this respect, the box would have a negative regulatory role in XOTX2, and a positive one in XOTX5b.

We also investigated whether the difference in the synergy of XOTX5b and XOTX2 with XNRL on the rhodopsin promoter may be due to differences in their interaction with XNRL. We found that MYC-XOTX5b had a significantly greater affinity toward GST-XNRL than MYC-XOTX2, MYC-XOTX5bMut3 and MYC-OTD/box2 (all with an XOTX2-type box) or MYC-XOTX2Δ, MYC-XOTX5bΔ, and MYC-OTD (all lacking a box); instead, MYC-XOTX2Mut3 and MYC-OTD/box5b (with a XOTX5b-type box) were not significantly different from XOTX5b in this respect. Therefore, while the box is not an essential determinant for XOTX/OTD versus XNRL interaction, it seems to modulate this interaction in a way that is quite consistent with the roles of XOTX2 and XOTX5b in frog retinogenesis [12] and with the results on rhodopsin promoter activation [37] (and present data).

On the whole, our results show that XOTX proteins are pivotal in regulating retinal cell fate. Although their effects on cell fate may appear limited, and not all transfected progenitors are turned into bipolar cells or photoreceptors, they are statistically significant. Failure to address all transfected cells to a single and specific fate may be due to differences in their time of cell cycle exit, which may underlie the diverse competence of progenitors; it may be significant, in this respect, that the effect of *Xotx5b *in enhancing photoreceptor fate is potentiated when co-transfected with *X-gadd45γ*, which promotes cell cycle exit [47]. Besides, retinal cell fate commitment and differentiation are, at the molecular level, the result of a multifactorial process [6,16,17], and, therefore, other factors may contribute to the action of either XOTX2 or XOTX5b in addressing retinal cells to their proper neuronal fate.

## Conclusion

We have provided *in vivo *and *in vitro *evidence for the different biochemical activities of XOTX/OTD proteins in the *Xenopus *retina, due to the presence/absence of the RS box. We suggest that the RS box allows XOTX2 and XOTX5b proteins to appropriately target gene sets involved in bipolar and photoreceptor cell specification, respectively. This is particularly significant since OTX/CRX/OTD proteins are able to bind *in vitro *to the same consensus sequence, TAATCC/T [33,53,54], and yet they have significantly different effects in the *Xenopus *retina; this is reminiscent of many *Hox *gene products, which also have largely overlapping DNA binding abilities but perform very specific and different developmental functions (reviewed in [55,56]). While still relatively little is known about what confers *in vivo *targeting specificity on homeodomain containing factors, our data show that the RS box of XOTX2 and XOTX5b is an essential and major domain of their functioning *in vivo *and is involved in providing such specificity in the developing frog retina.

## Methods

### *Xenopus laevis *embryos

*Xenopus *embryos were obtained and staged as previously described [16]. All protocols involving the use of animals were approved by the Bioethical Committee of Pisa University.

### DNA constructs

The main constructs used in this study are shown in Figure 1. pCS2Xotx2 and pCS2Xotx5b wild-type constructs were described in Viczian *et al*. [12]. pCS2Xotx5bMut1, pCS2Xotx5bMut2 and pCS2Xotx5bMut3 were generated by *in vitro *mutagenesis from pCS2Xotx5b, converting the initial STGQAKPR sequence of amino acids 100-107 of XOTX5b to generate mutant constructs as shown in Figure 1. Similarly, pCS2Xotx2Mut3 was obtained from pCS2Xotx2. pCS2otd was obtained by PCR cloning of the *otd *coding region plus 50 nucleotides (nt) of the 5' UTR and 24 nt of the 3' UTR into pCS2+; fragments were amplified from an *otd *plasmid (kindly provided by Dr Antonio Simeone), and cloned into the *Eco*RI site of pCS2+. The chimeric pCS2otd/Xotx2 is an in-frame fusion encoding amino acids 1–96 of OTD and amino acids 62–288 of XOTX2, plus 50 nt of the 5' UTR of *otd *and 4 nt of the 3' UTR of *Xotx2*; the chimeric pCS2otd/Xotx5b is an in-frame fusion encoding amino acids 1–96 of OTD and amino acids 62–290 of XOTX5b, plus 50 nt of the 5' UTR of *otd *and 25 nt of the 3' UTR of *Xotx5b*. The pCS2Xotx2Δ and pCS2Xotx5bΔ deletion constructs correspond to pCS2Xotx2 and pCS2Xotx5b, respectively, except that the regions encoding amino acids 100–109 of XOTX2 and amino acids 100–107 of XOTX5b were removed by site directed mutagenesis. pCS2otd/box2 and pCS2otd/box5b correspond to pCS2otd, but have an insertion encoding amino acids 100–109 of XOTX2 and 100–107 of XOTX5b, respectively, replacing amino acids 132–137 of OTD.

pCS2Myc-Xotx2, pCS2Myc-Xotx5b, pCS2Myc-Xotx2Mut3, pCS2Myc-Xotx5bMut3, pCS2Myc-Xotx2Δ, pCS2Myc-Xotx5bΔ, pCS2Myc-otd, pCS2Myc-otd/box2, pCS2Myc-otd/box5b, and pCS2-NLS-Myc-otd were prepared by PCR cloning from the parental *Xotx2*, *Xotx5b*, *otd *or chimeric plasmids into pCS2Myc and pCS2-NLS-Myc vectors.

*Xnrl *full-length cDNA was cloned by RT-PCR from stage 42 *Xenopus *embryo RNA; the GST-Xnrl fusion construct was obtained by in frame PCR cloning of the *Xnrl *coding region into the pYEX vector (a modified pGEX 2TK, a kind gift of Dr Luciana Dente). All constructs were verified by sequencing.

### Lipofections

Lipofections were performed at stages 17–18 as described in Poggi *et al*. [16]. At stage 42, embryos were fixed in 4% paraformaldehyde for 1 h at room temperature, sunk in 20% sucrose overnight at 4°C and cryostat sectioned (12 μm). Samples were rehydrated with two washes of 1× phosphate-buffered saline (PBS) for 5 minutes, incubated in 1 μg/ml Hoechst to label nuclei and mounted in Aqua Polymount (Polysciences, Inc., Warrington, PA, U.S.A). Lipofected cells were scored by GFP fluorescence and assigned to the different cell types on the basis of their position within layers and their morphology; their identity was confirmed by molecular marker analysis (Additional file 3). Statistical analysis on cell frequencies was performed by means of one-way ANOVA and Tukey-Kramer multiple comparison test.

### *In situ *hybridization, immunostaining and immunofluorescence

*In situ *hybridization on sections was performed as previously described [57]; immunostaining on sections as described in [12,47]. Probes used for *in situ *hybridizations were: *XIRBP *for photoreceptors [58]; *Xhermes *for ganglion cells [59]; *Xprox1 *for horizontal cells [11]; and *Xvsx1 *for bipolar cells [48]. To identify amacrine cells we used anti-5-HT, anti-GABA and anti-Tyrosine Hydroxylase, all purchased from DiaSorin (Saluggia, Italy). The anti-XOTX2 and anti-XOTX5b antibodies were described in Decembrini *et al*. [47].

### GST-XNRL protein production and purification

GST-fusion proteins were expressed in *Escherichia coli *BL21 upon transformation with appropriate constructs. Cultures were grown to mid-log phase (A_600 _= 0.7) in LB medium at 37°C, induced with 1.0 mM isopropyl thio-β-D-galactopiranoside, and grown for an additional 4 h at 32°C. Culture (50 ml) was centrifuged at 4,000 rpm for 15 minutes, resuspended in ice cold PBS and lysed on ice. After addition of lysozyme (200 μg/ml), 10 mM DTT (in AcONa 10 mM pH 5.2), protease inhibitor mix (2 mM AEBSF, 1 mM EDTA, 130 μM bestatin, 14 μM E-64, 1 μM leupeptin, 0.3 μM aprotinin; final concentrations; Sigma (S. Louis, MO, U.S.A.)), the mixture was left on ice for 30 minutes. Then 1% (v/v) Triton X-100, 10 mM MgCl_2_, and 100 μg/ml DNase (final concentrations) were added; the mixture was left on ice for further 30 minutes and then centrifuged at 4°C, 14,000 rpm for 20 minutes.

Glutathione Sepharose 4B resin (100 μl) (Amersham, GE Healthcare, Little Chalfont, Buckinghamshire, U.K.) was used for each experiment and control. Resin was washed 3 times with ice cold PBS and centrifuged at 2,500 rpm for 1 minute following each wash. BL21 extract was then incubated with the resin for 1 h at 4°C on a shaker. Then three washings were carried out as above. Finally, the resin was soaked in a solution of 3% (w/v) bovine serum albumin in PBS to achieve blocking, and left at 4°C overnight.

### Cell transfection and pull-down assay

HEK 293T cells were cultured in Dulbecco's modified Eagle's medium (Gibco/Invitrogen, Grand Island, NY, U.S.A.) supplemented with 10% (v/v) fetal bovine serum (GIBCO). Transfections were performed using Lipofectamine 2000 (Invitrogen, Grand Island, NY, U.S.A.). Following a 48 h incubation at 37°C and 5% CO_2_, cells were washed with ice-cold PBS and lysed with 100 μl ice-cold lysis buffer (1% (v/v) Triton X-100, 1 mM phenylmethylsulfonyl fluoride, 50 mM HEPES pH 7.5, 150 mM NaCl, 1% glycerol, 1.5 mM MgCl_2_, 5 mM EGTA, 1 mM Na_3_VO_4 _and protease inhibitor cocktail (Sigma)). After 30 minutes incubation on ice, lysates were cleared by centrifugation for 40 minutes at 14,000 g and 4°C. After protein quantification of the extracts (Bradford assay), Myc fusion proteins were purified on anti-cMyc antibody agarose beads (Clontech n. 631208, Clontech, Mountain View, CA, U.S.A.), quantified again and subsequently incubated with Glutathione-Sepharose-bound GST-XNRL or GST alone in the binding buffer (0.1% Triton X-100, 50 mM HEPES pH 7.5, 150 mM NaCl, 1% glycerol, 1.5 mM MgCl_2_, 5 mM EGTA) overnight at 4°C on a shaker; then washed three times and finally denatured with loading buffer for 5 minutes at 95°C.

### Western blotting

Protein samples were loaded onto a 12% polyacrylamide gel for size separation. Subsequently, proteins were transferred to Immobilon-P Tranfer membrane (Millipore, Billerica, MA, U.S.A.) by electroblotting for 1–2 h. Blots were blocked for 1 h using 5% nonfat dry milk in TBS-T (10 mM Tris/HCl, pH 8.0, 150 mM NaCl, 0.05% (v/v) Tween-20 (Sigma)). Monoclonal primary anti-MYC antibody (Sigma) (dilution 1:500) and secondary anti mouse IgG (peroxidase conjugate; Sigma; 1:10,000) were used to detect MYC-tagged proteins. Filters were incubated for 1 h at room temperature for each antibody, and then washed three times with TBS-T to remove excess antibody. The SuperSignal West Pico Chemiluminescent Substrate (Pierce, Rockford, IL, U.S.A.) was used to visualize immunoreactive bands by exposure to Amersham Hyperfilm. Samples from two independent experiments were analyzed.

### Cell transfection and reporter assays

HEK 293T cells were co-transfected in 24-well plates with a total of 600 ng of DNA using Lipofectamine. XOP-GFP construct (400 ng) [50] was added to each well, along with various combinations of 100 ng of pCS2Xotx2, pCS2Xotx2Mut3, pCS2Xotx2Δ, pCS2Xotx5b, pCS2Xotx5bMut3, pCS2Xotx5bΔ, pCS2otd, pCS2otd/Xotx2, pCS2otd/Xotx5b, pCS2otd/box2, pCS2otd/box5b, pCS2Xnrl, or empty pYEX expression constructs. GFP fluorescence was analyzed using flow cytometry. Fold activation was assumed as the ratio of the volume of fluorescence between each sample and the basal activation sample (XOP-GFP transfection alone), where the fluorescent volume is the fraction of GFP positive cells in the population multiplied by the mean fluorescence intensity [60]. Samples from at least three independent experiments were analyzed.

## Competing interests

The author(s) declare that they have no competing interests.

## Authors' contributions

MO performed most of the experiments, designed and generated many of the constructs used in this study and analyzed the data. FC contributed to conceive some of the experiments, performed some of the immunostaining experiments, analyzed the lipofection results and contributed to the writing of the paper. YL designed and prepared the mutant *Xotx5b *constructs. R-QE participated in the design of lipofection experiments and of some of the constructs. GB and RV conceived the study, participated in the design of most of the experiments and wrote the manuscript. All authors read and approved the final manuscript.

## Supplementary Material

Additional file 1 Raw data of the lipofection experiments with the *Xotx5bMut1*, *Xotx5bMut2*, *Xotx5bMut3*, *otd *and *otd/Xotx *constructs. Raw data of the lipofection experiments with the *Xotx5bMut1*, *Xotx5bMut2*, and *Xotx5bMut3 *constructs (summarized in Figure 2e), with the *otd *construct (summarized in Figure 4a) and with the *otd/Xotx *constructs (summarized in Figure 4b). Click here for file

Additional file 2 Results of the statistical ANOVA analysis on neuronal cell type frequency in lipofection experiments with *Xotx5bMut1*, *Xotx5bMut2*, and *Xotx5bMut3 *constructs. Results of the statistical ANOVA analysis on neuronal cell type frequency in lipofection experiments with *Xotx5bMut1*, *Xotx5bMut2*, and *Xotx5bMut3 *constructs (Figure 2e). Click here for file

Additional file 3 Expression of retinal markers used in this study to confirm neuron cell identities of lipofected cells. Lipofected GFP-positive cells (green) can be identified by using specific probes (all detected with Fast Red): **(a) **an *IRBP *probe identifies photoreceptors by *in situ *hybridization; **(b) ***in situ *hybridization with *Xprox1 *identifies horizontal cells (red); **(c) ***in situ *hybridization with *Xvsx1 *identifies bipolar cells (red); **(d) **a specific antibody identifies GABAergic amacrine cells (red); **(e) **a specific antibody identifies 5-HT positive amacrine cells (red); **(f) **a specific antibody identifies tyrosine hydroxylase (TH)-positive amacrine cells (red); **(g) ***in situ *hybridization with *Xhermes *identifies ganglion cells. Hoechst staining (in blue) identifies cell nuclei. Click here for file

Additional file 4 Raw data of the lipofection experiments with the *Xotx2Mut3 *construct. Raw data of the lipofection experiments with the *Xotx2Mut3 *construct (summarized in Figure 3a). Click here for file

Additional file 5 Results of the statistical ANOVA analysis on neuronal cell type frequency in lipofection experiments with the *Xotx2Mut3 *construct. Results of the statistical ANOVA analysis on neuronal cell type frequency in lipofection experiments with the *Xotx2Mut3 *construct (Figure 3a). Click here for file

Additional file 6 Raw data of the lipofection experiments with the *Xotx2Δ *and *Xotx5bΔ *constructs. Raw data of the lipofection experiments with the *Xotx2Δ *and *Xotx5bΔ *constructs (summarized in Figure 3f). Click here for file

Additional file 7 Results of the statistical ANOVA analysis on neuronal cell type frequency in lipofection experiments with the *Xotx *deletion constructs. Results of the statistical ANOVA analysis on neuronal cell type frequency in lipofection experiments with the *Xotx *deletion constructs (Figure 3f). Click here for file

Additional file 8 Results of the statistical ANOVA analysis on neuronal cell type frequency in lipofection experiments with the *otd/Xotx *constructs. Results of the statistical ANOVA analysis on neuronal cell type frequency in lipofection experiments with the *otd/Xotx *constructs (Figure 4b). Click here for file

Additional file 9 Raw data of the lipofection experiments with the *otd/box2 *and *otd/box5b *constructs. Raw data of the lipofection experiments with the *otd/box2 *and *otd/box5b *constructs (summarized in Figure 4c). Click here for file

Additional file 10 Results of the statistical ANOVA analysis on neuronal cell type frequency in lipofection experiments with the *otd/box2 *and *otd/box5b *constructs. Results of the statistical ANOVA analysis on neuronal cell type frequency in lipofection experiments with the *otd/box2 *and *otd/box5b *constructs (Figure 4c). Click here for file

Additional file 11 Raw data of the lipofection experiments with the NLS-Myc-*otd *construct. Raw data of the lipofection experiments with the NLS-Myc-*otd *construct (summarized in Figure 5c). Click here for file

Additional file 12 Raw data of the rhodopsin promoter transactivation assays. Raw data of the rhodopsin promoter transactivation assays summarized in Figure 6a. Click here for file

Additional file 13 Raw data of the GST-pull down experiments. Raw data of the GST-pull down experiments summarized in Figure 6b, c. Click here for file
