# Supplementary material for: A specific box switches the cell fate determining activity of XOTX2 and XOTX5b in the Xenopus retina
Source: Neural Dev. 2007 Jun 27;2:12. doi: 10.1186/1749-8104-2-12 (PMC1929070; doi:10.1186/1749-8104-2-12)
Supplement: Additional file 2 — Results of the statistical ANOVA analysis on neuronal cell type frequency in lipofection experiments with Xotx5bMut1, Xotx5bMut2, and Xotx5bMut3 constructs. Results of the statistical ANOVA analysis on neuronal cell type frequency in lipofection experiments with Xotx5bMut1, Xotx5bMut2, and Xotx5bMut3 constructs (Figure 2e). [file 1749-8104-2-12-S2.pdf]

Amacrine cells

One-way Analysis of Variance (ANOVA)

The P value is < 0.0001, considered extremely significant.
Variation among column means is significantly greater than expected by chance.

Tukey-Kramer Multiple Comparisons Test
If the value of q is greater than 4.140 then the P value is less than 0.05.

Comparison	Mean Difference	q		P value
GFP vs Xotx5b	4.408	4.886	*	P<0.05
GFP vs Xotx2	1.738	2.012	ns	P>0.05
GFP vs Xotx5b Mut3/1	3.486	3.926	ns	P>0.05
GFP vs Xotx5b Mut2/1	2.644	2.621	ns	P>0.05
GFP vs Xotx5b Mut1/4	6.631	7.083	***	P<0.001
Xotx5b vs Xotx2	-2.670	3.091	ns	P>0.05
Xotx5b vs Xotx5b Mut3/1	-0.9213	1.038	ns	P>0.05
Xotx5b vs Xotx5b Mut2/1	-1.764	1.749	ns	P>0.05
Xotx5b vs Xotx5b Mut1/4	2.223	2.375	ns	P>0.05
Xotx2 vs Xotx5b Mut3/1	1.749	2.060	ns	P>0.05
Xotx2 vs Xotx5b Mut2/1	0.9062	0.9300	ns	P>0.05
Xotx2 vs Xotx5b Mut1/4	4.893	5.441	**	P<0.01
Xotx5b Mut3/1 vs Xotx5b Mut2/1	-0.8426	0.8460	ns	P>0.05
Xotx5b Mut3/1 vs Xotx5b Mut1/4	3.144	3.409	ns	P>0.05
Xotx5b Mut2/1 vs Xotx5b Mut1/4	3.987	3.837	ns	P>0.05

Difference	Mean Difference	95% Confidence Interval From	To
GFP - Xotx5b	4.408	0.6731	8.142
GFP - Xotx2	1.738	-1.838	5.313
GFP - Xotx5b Mut3/1	3.486	-0.1894	7.162
GFP - Xotx5b Mut2/1	2.644	-1.532	6.819
GFP - Xotx5b Mut1/4	6.631	2.755	10.506
Xotx5b - Xotx2	-2.670	-6.246	0.9055
Xotx5b - Xotx5b Mut3/1	-0.9213	-4.597	2.754
Xotx5b - Xotx5b Mut2/1	-1.764	-5.939	2.411
Xotx5b - Xotx5b Mut1/4	2.223	-1.652	6.099
Xotx2 - Xotx5b Mut3/1	1.749	-1.765	5.263
Xotx2 - Xotx5b Mut2/1	0.9062	-3.128	4.940
Xotx2 - Xotx5b Mut1/4	4.893	1.171	8.616
Xotx5b Mut3/1 - Xotx5b Mut2/1	-0.8426	-4.965	3.280
Xotx5b Mut3/1 - Xotx5b Mut1/4	3.144	-0.6744	6.963
Xotx5b Mut2/1 - Xotx5b Mut1/4	3.987	-0.3149	8.289

Assumption test: Are the standard deviations of the groups equal?

ANOVA assumes that the data are sampled from populations with identical SDs. This assumption is tested using the method of Bartlett.

Bartlett statistic (corrected) = 4.035
The P value is 0.5444.
Bartlett's test suggests that the differences among the SDs is not significant.

Assumption test: Are the data sampled from Gaussian distributions?

ANOVA assumes that the data are sampled from populations that follow Gaussian distributions. This assumption is tested using the method Kolmogorov and Smirnov:

Group	KS	P Value	Passed normality test?
GFP	0.1229	>0.10	Yes
Xotx5b	0.2231	>0.10	Yes
Xotx2	0.1431	>0.10	Yes
Xotx5b Mut3/1	0.09203	>0.10	Yes
Xotx5b Mut2/1	0.1779	>0.10	Yes
Xotx5b Mut1/4	0.1899	>0.10	Yes

Intermediate calculations. ANOVA table

Source of variation	Degrees of freedom	Sum of squares	Mean square
Treatments (between columns)	5	369.57	73.914
Residuals (within columns)	81	988.88	12.208
Total	86	1358.5	

F = 6.054 =(MStreatment/MSresidual)

Summary of Data

Group	Number of Points	Mean	Standard Deviation	Standard Error of Mean	Median
GFP	15	16.218	3.790	0.9786	16.102
Xotx5b	15	11.810	3.601	0.9299	12.150
Xotx2	18	14.480	2.553	0.6017	14.538
Xotx5b Mut3/1	16	12.731	3.198	0.7995	12.641
Xotx5b Mut2/1	10	13.574	3.989	1.262	13.985
Xotx5b Mut1/4	13	9.587	4.070	1.129	10.227

Group	Minimum	Maximum	95% Confidence Interval From	To
GFP	9.524	22.609	14.118	18.317
Xotx5b	1.961	18.293	9.815	13.805
Xotx2	7.692	20.430	13.210	15.750
Xotx5b Mut3/1	7.895	19.298	11.028	14.435
Xotx5b Mut2/1	6.897	20.000	10.720	16.427
Xotx5b Mut1/4	0.000	15.126	7.127	12.046

* * *

Bipolar cells

One-way Analysis of Variance (ANOVA)

The P value is < 0.0001, considered extremely significant.
Variation among column means is significantly greater than expected by chance.

Tukey-Kramer Multiple Comparisons Test
If the value of q is greater than 4.140 then the P value is less than 0.05.

Comparison	Mean Difference	q		P value
GFP vs Xotx5b	-1.456	1.606	ns	P>0.05
GFP vs Xotx2	-9.206	10.606	***	P<0.001
GFP vs Xotx5b Mut3/1	-11.050	12.383	***	P<0.001
GFP vs Xotx5b Mut2/1	-8.149	8.039	***	P<0.001
GFP vs Xotx5b Mut1/4	-5.878	6.248	***	P<0.001
Xotx5b vs Xotx2	-7.751	8.929	***	P<0.001
Xotx5b vs Xotx5b Mut3/1	-9.594	10.751	***	P<0.001
Xotx5b vs Xotx5b Mut2/1	-6.693	6.603	***	P<0.001
Xotx5b vs Xotx5b Mut1/4	-4.422	4.700	*	P<0.05
Xotx2 vs Xotx5b Mut3/1	-1.843	2.161	ns	P>0.05
Xotx2 vs Xotx5b Mut2/1	1.058	1.080	ns	P>0.05
Xotx2 vs Xotx5b Mut1/4	3.328	3.683	ns	P>0.05
Xotx5b Mut3/1 vs Xotx5b Mut2/1	2.901	2.899	ns	P>0.05
Xotx5b Mut3/1 vs Xotx5b Mut1/4	5.172	5.578	**	P<0.01
Xotx5b Mut2/1 vs Xotx5b Mut1/4	2.271	2.174	ns	P>0.05

Difference	Mean Difference	95% Confidence Interval From	To
GFP - Xotx5b	-1.456	-5.209	2.297
GFP - Xotx2	-9.206	-12.800	-5.613
GFP - Xotx5b Mut3/1	-11.050	-14.744	-7.356
GFP - Xotx5b Mut2/1	-8.149	-12.345	-3.953
GFP - Xotx5b Mut1/4	-5.878	-9.773	-1.983
Xotx5b - Xotx2	-7.751	-11.344	-4.157
Xotx5b - Xotx5b Mut3/1	-9.594	-13.288	-5.900
Xotx5b - Xotx5b Mut2/1	-6.693	-10.889	-2.497
Xotx5b - Xotx5b Mut1/4	-4.422	-8.317	-0.5276
Xotx2 - Xotx5b Mut3/1	-1.843	-5.375	1.688
Xotx2 - Xotx5b Mut2/1	1.058	-2.996	5.112
Xotx2 - Xotx5b Mut1/4	3.328	-0.4126	7.069
Xotx5b Mut3/1 - Xotx5b Mut2/1	2.901	-1.242	7.044
Xotx5b Mut3/1 - Xotx5b Mut1/4	5.172	1.334	9.009
Xotx5b Mut2/1 - Xotx5b Mut1/4	2.271	-2.053	6.594

Assumption test: Are the standard deviations of the groups equal?

ANOVA assumes that the data are sampled from populations with identical SDs. This assumption is tested using the method of Bartlett.

Bartlett statistic (corrected) = 6.676
The P value is 0.2459.
Bartlett's test suggests that the differences among the SDs is not significant.

Assumption test: Are the data sampled from Gaussian distributions?

ANOVA assumes that the data are sampled from populations that follow Gaussian distributions. This assumption is tested using the method Kolmogorov and Smirnov:

**Group**	**KS**	**P Value**	**Passed normality test?**
GFP	0.1836	>0.10	Yes
Xotx5b	0.1675	>0.10	Yes
Xotx2	0.2534	>0.10	Yes
Xotx5b Mut3/1	0.1620	>0.10	Yes
Xotx5b Mut2/1	0.1865	>0.10	Yes
Xotx5b Mut1/4	0.1854	>0.10	Yes

Intermediate calculations. ANOVA table

Source of variation	Degrees of freedom	Sum of squares	Mean square
Treatments (between columns)	5	1489.2	297.83
Residuals (within columns)	81	998.68	12.329
Total	86	2487.8	

F = 24.156 =(MStreatment/MSresidual)

Summary of Data

**Group**	**Number of Points**	**Mean**	**Standard Deviation**	**Standard Error of Mean**	**Median**
GFP	15	29.028	2.034	0.5251	28.571
Xotx5b	15	30.484	3.622	0.9352	31.148
Xotx2	18	38.234	3.532	0.8326	38.152
Xotx5b Mut3/1	16	40.077	3.871	0.9678	41.090
Xotx5b Mut2/1	10	37.176	4.050	1.281	36.826
Xotx5b Mut1/4	13	34.906	3.793	1.052	35.106

			**95% Confidence Interval**	
**Group**	**Minimum**	**Maximum**	**From**	**To**
GFP	26.667	33.333	27.901	30.154
Xotx5b	24.735	35.000	28.478	32.490
Xotx2	34.409	50.000	36.477	39.991
Xotx5b Mut3/1	33.824	45.455	38.015	42.140
Xotx5b Mut2/1	32.632	44.828	34.280	40.073
Xotx5b Mut1/4	25.210	39.773	32.614	37.198

* * *

Photoreceptors

One-way Analysis of Variance (ANOVA)

The P value is < 0.0001, considered extremely significant.
Variation among column means is significantly greater than expected by chance.

Tukey-Kramer Multiple Comparisons Test
If the value of q is greater than 4.140 then the P value is less than 0.05.

Comparison	Mean Difference	q		P value
GFP vs Xotx5b	-9.406	10.855	***	P<0.001
GFP vs Xotx2	6.430	7.750	***	P<0.001
GFP vs Xotx5b Mut3/1	4.346	5.096	**	P<0.01
GFP vs Xotx5b Mut2/1	3.028	3.125	ns	P>0.05
GFP vs Xotx5b Mut1/4	-4.060	4.515	*	P<0.05
Xotx5b vs Xotx2	15.835	19.088	***	P<0.001
Xotx5b vs Xotx5b Mut3/1	13.752	16.124	***	P<0.001
Xotx5b vs Xotx5b Mut2/1	12.434	12.834	***	P<0.001
Xotx5b vs Xotx5b Mut1/4	5.346	5.945	**	P<0.01
Xotx2 vs Xotx5b Mut3/1	-2.084	2.556	ns	P>0.05
Xotx2 vs Xotx5b Mut2/1	-3.402	3.635	ns	P>0.05
Xotx2 vs Xotx5b Mut1/4	-10.489	12.144	***	P<0.001
Xotx5b Mut3/1 vs Xotx5b Mut2/1	-1.318	1.378	ns	P>0.05
Xotx5b Mut3/1 vs Xotx5b Mut1/4	-8.406	9.486	***	P<0.001
Xotx5b Mut2/1 vs Xotx5b Mut1/4	-7.088	7.101	***	P<0.001

Difference	Mean Difference	95% Confidence Interval From	To
GFP - Xotx5b	-9.406	-12.993	-5.819
GFP - Xotx2	6.430	2.996	9.864
GFP - Xotx5b Mut3/1	4.346	0.8155	7.876
GFP - Xotx5b Mut2/1	3.028	-0.9824	7.038
GFP - Xotx5b Mut1/4	-4.060	-7.782	-0.3373
Xotx5b - Xotx2	15.835	12.401	19.270
Xotx5b - Xotx5b Mut3/1	13.752	10.221	17.282
Xotx5b - Xotx5b Mut2/1	12.434	8.423	16.444
Xotx5b - Xotx5b Mut1/4	5.346	1.624	9.068
Xotx2 - Xotx5b Mut3/1	-2.084	-5.459	1.291
Xotx2 - Xotx5b Mut2/1	-3.402	-7.276	0.4725
Xotx2 - Xotx5b Mut1/4	-10.489	-14.065	-6.914
Xotx5b Mut3/1 - Xotx5b Mut2/1	-1.318	-5.278	2.642
Xotx5b Mut3/1 - Xotx5b Mut1/4	-8.406	-12.074	-4.738
Xotx5b Mut2/1 - Xotx5b Mut1/4	-7.088	-11.220	-2.956

Assumption test: Are the standard deviations of the groups equal?

ANOVA assumes that the data are sampled from populations with identical SDs. This assumption is tested using the method of Bartlett.

Bartlett statistic (corrected) = 20.100
The P value is 0.0012.
Bartlett's test suggests that the differences among the SDs is very significant.
Since ANOVA assumes populations with equal SDs, you should consider transforming your data (reciprocal or log) or selecting a nonparametric test.

Assumption test: Are the data sampled from Gaussian distributions?

ANOVA assumes that the data are sampled from populations that follow Gaussian distributions. This assumption is tested using the method Kolmogorov and Smirnov:

**Group**	**KS**	**P Value**	**Passed normality test?**
GFP	0.08800	>0.10	Yes
Xotx5b	0.2178	>0.10	Yes
Xotx2	0.1602	>0.10	Yes
Xotx5b Mut3/1	0.2448	>0.10	Yes
Xotx5b Mut2/1	0.2367	>0.10	Yes
Xotx5b Mut1/4	0.1723	>0.10	Yes

Intermediate calculations. ANOVA table

Source of variation	Degrees of freedom	Sum of squares	Mean square
Treatments (between columns)	5	2673.9	534.78
Residuals (within columns)	81	912.27	11.263
Total	86	3586.2	

F = 47.482 =(MStreatment/MSresidual)

Summary of Data

**Group**	**Number of Points**	**Mean**	**Standard Deviation**	**Standard Error of Mean**	**Median**
GFP	15	30.393	2.861	0.7388	30.000
Xotx5b	15	39.799	5.172	1.335	38.000
Xotx2	18	23.964	2.278	0.5370	23.785
Xotx5b Mut3/1	16	26.047	1.865	0.4663	26.471
Xotx5b Mut2/1	10	27.365	4.356	1.377	29.217
Xotx5b Mut1/4	13	34.453	3.055	0.8474	35.498

**Group**	**Minimum**	**Maximum**	**95% Confidence Interval From**	**To**
GFP	26.271	35.766	28.809	31.978
Xotx5b	34.756	51.852	36.935	42.663
Xotx2	19.355	27.404	22.830	25.097
Xotx5b Mut3/1	21.635	28.788	25.054	27.041
Xotx5b Mut2/1	21.429	33.333	24.250	30.481
Xotx5b Mut1/4	30.120	40.000	32.607	36.299

*   *   *

Ganglion cells

One-way Analysis of Variance (ANOVA)

The P value is 0.0638, considered not quite significant.
Variation among column means is not significantly greater than expected by chance.

Tukey-Kramer Multiple Comparisons Test

If the value of q is greater than 4.140 then the P value is less than 0.05.

Comparison	Mean Difference	q		P value
GFP vs Xotx5b	4.113	4.066	ns	P>0.05
GFP vs Xotx2	2.634	2.719	ns	P>0.05
GFP vs Xotx5b Mut3/1	1.983	1.992	ns	P>0.05
GFP vs Xotx5b Mut2/1	0.2040	0.1804	ns	P>0.05
GFP vs Xotx5b Mut1/4	2.317	2.207	ns	P>0.05
Xotx5b vs Xotx2	-1.479	1.527	ns	P>0.05
Xotx5b vs Xotx5b Mut3/1	-2.130	2.139	ns	P>0.05
Xotx5b vs Xotx5b Mut2/1	-3.909	3.456	ns	P>0.05
Xotx5b vs Xotx5b Mut1/4	-1.796	1.711	ns	P>0.05
Xotx2 vs Xotx5b Mut3/1	-0.6505	0.6833	ns	P>0.05
Xotx2 vs Xotx5b Mut2/1	-2.430	2.224	ns	P>0.05
Xotx2 vs Xotx5b Mut1/4	-0.3171	0.3144	ns	P>0.05
Xotx5b Mut3/1 vs Xotx5b Mut2/1	-1.779	1.593	ns	P>0.05
Xotx5b Mut3/1 vs Xotx5b Mut1/4	0.3334	0.3223	ns	P>0.05
Xotx5b Mut2/1 vs Xotx5b Mut1/4	2.113	1.813	ns	P>0.05

Difference	Mean Difference	95% Confidence Interval From	To
GFP - Xotx5b	4.113	-0.07486	8.301
GFP - Xotx2	2.634	-1.376	6.644
GFP - Xotx5b Mut3/1	1.983	-2.139	6.105
GFP - Xotx5b Mut2/1	0.2040	-4.478	4.886
GFP - Xotx5b Mut1/4	2.317	-2.029	6.663
Xotx5b - Xotx2	-1.479	-5.489	2.530
Xotx5b - Xotx5b Mut3/1	-2.130	-6.252	1.992
Xotx5b - Xotx5b Mut2/1	-3.909	-8.591	0.7732
Xotx5b - Xotx5b Mut1/4	-1.796	-6.142	2.550
Xotx2 - Xotx5b Mut3/1	-0.6505	-4.591	3.290
Xotx2 - Xotx5b Mut2/1	-2.430	-6.953	2.094
Xotx2 - Xotx5b Mut1/4	-0.3171	-4.492	3.857
Xotx5b Mut3/1 - Xotx5b Mut2/1	-1.779	-6.403	2.844
Xotx5b Mut3/1 - Xotx5b Mut1/4	0.3334	-3.949	4.616
Xotx5b Mut2/1 - Xotx5b Mut1/4	2.113	-2.711	6.937

Assumption test: Are the standard deviations of the groups equal?

ANOVA assumes that the data are sampled from populations with identical SDs. This assumption is tested using the method of Bartlett.

Bartlett statistic (corrected) = 4.566
The P value is 0.4711.
Bartlett's test suggests that the differences among the SDs is not significant.

Assumption test: Are the data sampled from Gaussian distributions?

ANOVA assumes that the data are sampled from populations that follow Gaussian distributions. This assumption is tested using the method Kolmogorov and Smirnov:

Group	KS	P Value	Passed normality test?
GFP	0.2191	>0.10	Yes
Xotx5b	0.1607	>0.10	Yes
Xotx2	0.1468	>0.10	Yes
Xotx5b Mut3/1	0.1222	>0.10	Yes
Xotx5b Mut2/1	0.1787	>0.10	Yes
Xotx5b Mut1/4	0.1387	>0.10	Yes

Intermediate calculations. ANOVA table

Source of variation	Degrees of freedom	Sum of squares	Mean square
Treatments (between columns)	5	167.76	33.553
Residuals (within columns)	81	1243.6	15.353
Total	86	1411.4	

F = 2.185 =(MStreatment/MSresidual)

Summary of Data

Group	Number of Points	Mean	Standard Deviation	Standard Error of Mean	Median
GFP	15	15.242	3.480	0.8985	14.365
Xotx5b	15	11.129	3.422	0.8837	10.448
Xotx2	18	12.608	4.061	0.9571	12.528
Xotx5b Mut3/1	16	13.259	3.823	0.9557	12.817
Xotx5b Mut2/1	10	15.038	5.624	1.779	15.733
Xotx5b Mut1/4	13	12.925	3.239	0.8983	12.766

Group	Minimum	Maximum	95% Confidence Interval From	To
GFP	11.429	23.810	13.315	17.169
Xotx5b	3.704	16.260	9.233	13.024
Xotx2	6.452	21.739	10.589	14.628
Xotx5b Mut3/1	6.667	19.663	11.222	15.295
Xotx5b Mut2/1	4.762	22.857	11.015	19.061
Xotx5b Mut1/4	8.081	17.647	10.968	14.883

* * *

Horizontal cells

One-way Analysis of Variance (ANOVA)

The P value is 0.0294, considered significant.
Variation among column means is significantly greater than expected by chance.

Tukey-Kramer Multiple Comparisons Test

If the value of q is greater than 4.140 then the P value is less than 0.05.

Comparison	Mean Difference	q		P value
GFP vs Xotx5b	2.341	2.502	ns	P>0.05
GFP vs Xotx2	-1.595	1.781	ns	P>0.05
GFP vs Xotx5b Mut3/1	1.234	1.340	ns	P>0.05
GFP vs Xotx5b Mut2/1	2.273	2.173	ns	P>0.05
GFP vs Xotx5b Mut1/4	0.9899	1.020	ns	P>0.05
Xotx5b vs Xotx2	-3.936	4.394	*	P<0.05
Xotx5b vs Xotx5b Mut3/1	-1.107	1.202	ns	P>0.05
Xotx5b vs Xotx5b Mut2/1	-0.06787	0.06489	ns	P>0.05
Xotx5b vs Xotx5b Mut1/4	-1.351	1.391	ns	P>0.05
Xotx2 vs Xotx5b Mut3/1	2.829	3.214	ns	P>0.05
Xotx2 vs Xotx5b Mut2/1	3.868	3.828	ns	P>0.05
Xotx2 vs Xotx5b Mut1/4	2.585	2.772	ns	P>0.05
Xotx5b Mut3/1 vs Xotx5b Mut2/1	1.039	1.006	ns	P>0.05
Xotx5b Mut3/1 vs Xotx5b Mut1/4	-0.2439	0.2550	ns	P>0.05
Xotx5b Mut2/1 vs Xotx5b Mut1/4	-1.283	1.191	ns	P>0.05

Difference	Mean Difference	95% Confidence Interval From	To
GFP - Xotx5b	2.341	-1.532	6.213
GFP - Xotx2	-1.595	-5.302	2.112
GFP - Xotx5b Mut3/1	1.234	-2.577	5.045
GFP - Xotx5b Mut2/1	2.273	-2.056	6.602
GFP - Xotx5b Mut1/4	0.9899	-3.028	5.008
Xotx5b - Xotx2	-3.936	-7.643	-0.2283
Xotx5b - Xotx5b Mut3/1	-1.107	-4.918	2.704
Xotx5b - Xotx5b Mut2/1	-0.06787	-4.397	4.261
Xotx5b - Xotx5b Mut1/4	-1.351	-5.369	2.668
Xotx2 - Xotx5b Mut3/1	2.829	-0.8147	6.472
Xotx2 - Xotx5b Mut2/1	3.868	-0.3147	8.050
Xotx2 - Xotx5b Mut1/4	2.585	-1.275	6.445
Xotx5b Mut3/1 - Xotx5b Mut2/1	1.039	-3.236	5.314
Xotx5b Mut3/1 - Xotx5b Mut1/4	-0.2439	-4.203	3.716
Xotx5b Mut2/1 - Xotx5b Mut1/4	-1.283	-5.743	3.178

Assumption test: Are the standard deviations of the groups equal?

ANOVA assumes that the data are sampled from populations with identical SDs. This assumption is tested using the method of Bartlett.

Bartlett statistic (corrected) = 7.089
The P value is 0.2141.
Bartlett's test suggests that the differences among the SDs is not significant.

Assumption test: Are the data sampled from Gaussian distributions?

ANOVA assumes that the data are sampled from populations that follow Gaussian distributions. This assumption is tested using the method Kolmogorov and Smirnov:

Group	KS	P Value	Passed normality test?
GFP	0.1247	>0.10	Yes
Xotx5b	0.1306	>0.10	Yes
Xotx2	0.2069	>0.10	Yes
Xotx5b Mut3/1	0.2148	>0.10	Yes
Xotx5b Mut2/1	0.2246	>0.10	Yes
Xotx5b Mut1/4	0.1091	>0.10	Yes

Intermediate calculations. ANOVA table

Source of variation	Degrees of freedom	Sum of squares	Mean square
Treatments (between columns)	5	172.90	34.581
Residuals (within columns)	81	1063.1	13.124
Total	86	1236.0	

F = 2.635 =(MStreatment/MSresidual)

Summary of Data

Group	Number of Points	Mean	Standard Deviation	Standard Error of Mean	Median
GFP	15	9.119	3.194	0.8247	8.571
Xotx5b	15	6.779	2.891	0.7464	6.504
Xotx2	18	10.714	4.979	1.173	10.148
Xotx5b Mut3/1	16	7.886	3.590	0.8974	6.930
Xotx5b Mut2/1	10	6.847	2.915	0.9218	6.171
Xotx5b Mut1/4	13	8.129	3.057	0.8478	8.000

Group	Minimum	Maximum	95% Confidence Interval From	To
GFP	4.762	15.254	7.350	10.888
Xotx5b	1.724	12.500	5.178	8.380
Xotx2	3.158	25.806	8.238	13.190
Xotx5b Mut3/1	2.941	15.909	5.973	9.798
Xotx5b Mut2/1	4.286	13.793	4.761	8.932
Xotx5b Mut1/4	2.564	13.253	6.282	9.977

* * *
