# Supplementary figures and images for: A specific box switches the cell fate determining activity of XOTX2 and XOTX5b in the Xenopus retina
Source: Neural Dev. 2007 Jun 27;2:12. doi: 10.1186/1749-8104-2-12 (PMC1929070; doi:10.1186/1749-8104-2-12)

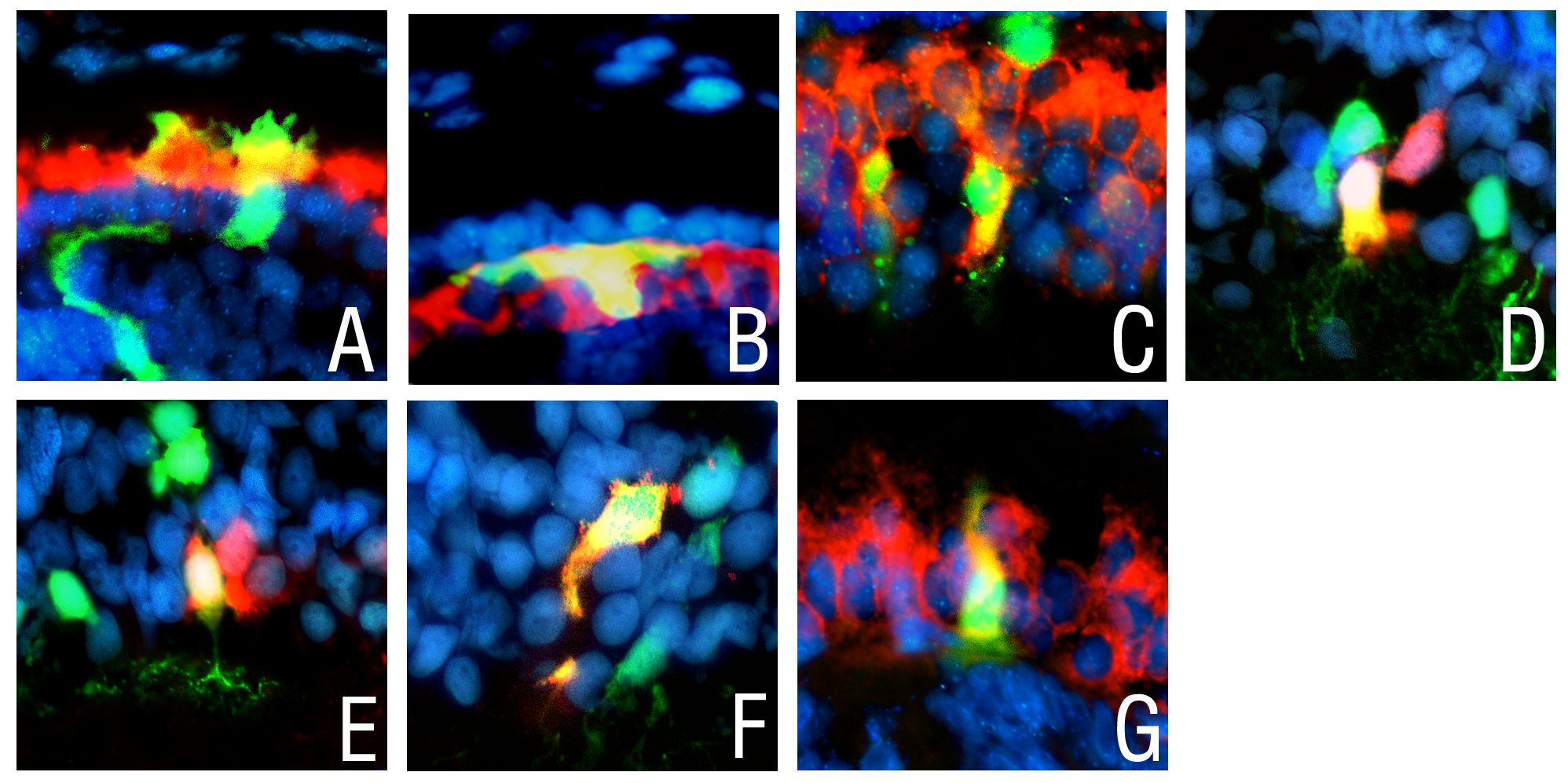

Supplement: Additional file 3 — Expression of retinal markers used in this study to confirm neuron cell identities of lipofected cells. Lipofected GFP-positive cells (green) can be identified by using specific probes (all detected with Fast Red): (a) an IRBP probe identifies photoreceptors by in situ hybridization; (b) in situ hybridization with Xprox1 identifies horizontal cells (red); (c) in situ hybridization with Xvsx1 identifies bipolar cells (red); (d) a specific antibody identifies GABAergic amacrine cells (red); (e) a specific antibody identifies 5-HT positive amacrine cells (red); (f) a specific antibody identifies tyrosine hydroxylase (TH)-positive amacrine cells (red); (g) in situ hybridization with Xhermes identifies ganglion cells. Hoechst staining (in blue) identifies cell nuclei. [file 1749-8104-2-12-S3.tiff]
