# Supplementary material for: A specific box switches the cell fate determining activity of XOTX2 and XOTX5b in the Xenopus retina
Source: Neural Dev. 2007 Jun 27;2:12. doi: 10.1186/1749-8104-2-12 (PMC1929070; doi:10.1186/1749-8104-2-12)
Supplement: Additional file 5 — Results of the statistical ANOVA analysis on neuronal cell type frequency in lipofection experiments with the Xotx2Mut3 construct. Results of the statistical ANOVA analysis on neuronal cell type frequency in lipofection experiments with the Xotx2Mut3 construct (Figure 3a). [file 1749-8104-2-12-S5.pdf]

Amacrin cells

## One-way Analysis of Variance (ANOVA)

The P value is 0.2551, considered not significant.
Variation among column means is not significantly greater than expected by chance.

### Post tests

Post tests were not calculated because the P value was greater than 0.05.

### Assumption test: Are the standard deviations of the groups equal?

ANOVA assumes that the data are sampled from populations with identical SDs. This assumption is tested using the method of Bartlett.

Bartlett statistic (corrected) = 7.751
The P value is 0.0515.
Bartlett's test suggests that the differences among the SDs is not quite significant.

### Assumption test: Are the data sampled from Gaussian distributions?

ANOVA assumes that the data are sampled from populations that follow Gaussian distributions. This assumption is tested using the method Kolmogorov and Smirnov:

| **Group** | **KS** | **P Value** | **Passed normality test?** |
|---|---|---|---|
| GFP | 0.3467 | >0.10 | Yes |
| Xotx5b | 0.1608 | >0.10 | Yes |
| Xotx2 | 0.1952 | >0.10 | Yes |
| Xotx2Mut3/1 | 0.1904 | >0.10 | Yes |

### Intermediate calculations. ANOVA table

| Source of variation | Degrees of freedom | Sum of squares | Mean square |
|---|---|---|---|
| Treatments (between columns) | 3 | 28.332 | 9.444 |
| Residuals (within columns) | 38 | 254.70 | 6.703 |
| Total | 41 | 283.03 | |

F = 1.409 =(MStreatment/MSresidual)

## Summary of Data

| **Group** | **Number of Points** | **Mean** | **Standard Deviation** | **Standard Error of Mean** | **Median** |
|---|---|---|---|---|---|
| GFP | 9 | 11.330 | 1.091 | 0.3637 | 10.949 |
| Xotx5b | 10 | 11.911 | 2.937 | 0.9286 | 11.556 |
| Xotx2 | 9 | 9.573 | 2.566 | 0.8552 | 9.821 |

| | | | | | |
|---|---|---|---|---|---|
| Xotx2Mut3/1 | 14 | 10.689 | 2.973 | 0.7946 | 9.830 |

| **Group** | **Minimum** | **Maximum** | **95% Confidence Interval From** | **To** |
|---|---|---|---|---|
| GFP | 10.448 | 14.063 | 10.492 | 12.169 |
| Xotx5b | 8.197 | 18.367 | 9.810 | 14.011 |
| Xotx2 | 6.383 | 13.462 | 7.601 | 11.545 |
| Xotx2Mut3/1 | 6.250 | 16.000 | 8.973 | 12.405 |

* * *

Bipolar cells

## One-way Analysis of Variance (ANOVA)

The P value is < 0.0001, considered extremely significant.
Variation among column means is significantly greater than expected by chance.

### Tukey-Kramer Multiple Comparisons Test

If the value of q is greater than 3.802 then the P value is less than 0.05.

| Comparison | Mean Difference | q | | P value |
|---|---|---|---|---|
| GFP vs Xotx5b | 2.403 | 1.986 | ns | P>0.05 |
| GFP vs Xotx2 | -8.810 | 7.094 | *** | P<0.001 |
| GFP vs Xotx2Mut3/1 | 1.431 | 1.271 | ns | P>0.05 |
| Xotx5b vs Xotx2 | -11.213 | 9.264 | *** | P<0.001 |
| Xotx5b vs Xotx2Mut3/1 | -0.9729 | 0.8920 | ns | P>0.05 |
| Xotx2 vs Xotx2Mut3/1 | 10.240 | 9.099 | *** | P<0.001 |

| Difference | Mean Difference | 95% Confidence Interval From | To |
|---|---|---|---|
| GFP - Xotx5b | 2.403 | -2.198 | 7.005 |
| GFP - Xotx2 | -8.810 | -13.531 | -4.089 |
| GFP - Xotx2Mut3/1 | 1.431 | -2.848 | 5.709 |
| Xotx5b - Xotx2 | -11.213 | -15.815 | -6.612 |
| Xotx5b - Xotx2Mut3/1 | -0.9729 | -5.119 | 3.174 |
| Xotx2 - Xotx2Mut3/1 | 10.240 | 5.962 | 14.519 |

### Assumption test: Are the standard deviations of the groups equal?

ANOVA assumes that the data are sampled from populations with identical SDs. This assumption is tested using the method of Bartlett.

Bartlett statistic (corrected) = 13.580
The P value is 0.0035.
Bartlett's test suggests that the differences among the SDs is very significant.
Since ANOVA assumes populations with equal SDs, you should consider transforming your data (reciprocal or log) or selecting a nonparametric test.

### Assumption test: Are the data sampled from Gaussian distributions?

ANOVA assumes that the data are sampled from populations that follow Gaussian distributions. This assumption is tested using the method Kolmogorov and Smirnov:

| Group | KS | P Value | Passed normality test? |
|---|---|---|---|

| | | | |
|---|---|---|---|
| GFP | 0.2287 | >0.10 | Yes |
| Xotx5b | 0.1773 | >0.10 | Yes |
| Xotx2 | 0.3268 | >0.10 | Yes |
| Xotx2Mut3/1 | 0.1750 | >0.10 | Yes |

Intermediate calculations. ANOVA table

| Source of variation | Degrees of freedom | Sum of squares | Mean square |
|---|---|---|---|
| Treatments (between columns) | 3 | 755.39 | 251.80 |
| Residuals (within columns) | 38 | 527.39 | 13.879 |
| Total | 41 | 1282.8 | |

F = 18.143 =(MStreatment/MSresidual)

Summary of Data

| **Group** | **Number of Points** | **Mean** | **Standard Deviation** | **Standard Error of Mean** | **Median** |
|---|---|---|---|---|---|
| GFP | 9 | 33.376 | 1.436 | 0.4787 | 33.333 |
| Xotx5b | 10 | 30.972 | 3.094 | 0.9783 | 31.140 |
| Xotx2 | 9 | 42.186 | 2.778 | 0.9261 | 43.802 |
| Xotx2Mut3/1 | 14 | 31.945 | 5.284 | 1.412 | 33.333 |

| **Group** | **Minimum** | **Maximum** | **95% Confidence Interval From** | **To** |
|---|---|---|---|---|
| GFP | 31.429 | 36.036 | 32.272 | 34.480 |
| Xotx5b | 25.758 | 34.483 | 28.760 | 33.185 |
| Xotx2 | 37.500 | 44.231 | 40.050 | 44.321 |
| Xotx2Mut3/1 | 22.826 | 37.778 | 28.895 | 34.996 |

* * *

Photoreceptors

## One-way Analysis of Variance (ANOVA)

The P value is < 0.0001, considered extremely significant.
Variation among column means is significantly greater than expected by chance.

### Tukey-Kramer Multiple Comparisons Test

If the value of q is greater than 3.802 then the P value is less than 0.05.

| Comparison | Mean Difference | q | | P value |
|---|---|---|---|---|
| GFP vs Xotx5b | -8.172 | 7.882 | *** | P<0.001 |
| GFP vs Xotx2 | 4.170 | 3.920 | * | P<0.05 |
| GFP vs Xotx2Mut3/1 | -6.288 | 6.522 | *** | P<0.001 |
| Xotx5b vs Xotx2 | 12.341 | 11.904 | *** | P<0.001 |
| Xotx5b vs Xotx2Mut3/1 | 1.884 | 2.016 | ns | P>0.05 |
| Xotx2 vs Xotx2Mut3/1 | -10.458 | 10.847 | *** | P<0.001 |

| Difference | Mean Difference | 95% Confidence Interval From | To |
|---|---|---|---|
| GFP - Xotx5b | -8.172 | -12.113 | -4.230 |
| GFP - Xotx2 | 4.170 | 0.1257 | 8.214 |
| GFP - Xotx2Mut3/1 | -6.288 | -9.953 | -2.623 |
| Xotx5b - Xotx2 | 12.341 | 8.400 | 16.283 |
| Xotx5b - Xotx2Mut3/1 | 1.884 | -1.668 | 5.436 |
| Xotx2 - Xotx2Mut3/1 | -10.458 | -14.123 | -6.792 |

### Assumption test: Are the standard deviations of the groups equal?

ANOVA assumes that the data are sampled from populations with identical SDs. This assumption is tested using the method of Bartlett.

Bartlett statistic (corrected) = 18.776
The P value is 0.0003.
Bartlett's test suggests that the differences among the SDs is extremely significant.
Since ANOVA assumes populations with equal SDs, you should consider transforming your data (reciprocal or log) or selecting a nonparametric test.

### Assumption test: Are the data sampled from Gaussian distributions?

ANOVA assumes that the data are sampled from populations that follow Gaussian distributions. This assumption is tested using the method Kolmogorov and Smirnov:

| Group | KS | P Value | Passed normality test? |
|---|---|---|---|

| | | | |
|---|---|---|---|
| GFP | 0.2934 | >0.10 | Yes |
| Xotx5b | 0.2542 | >0.10 | Yes |
| Xotx2 | 0.3169 | >0.10 | Yes |
| Xotx2Mut3/1 | 0.2103 | >0.10 | Yes |

Intermediate calculations. ANOVA table

| Source of variation | Degrees of freedom | Sum of squares | Mean square |
|---|---|---|---|
| Treatments (between columns) | 3 | 961.50 | 320.50 |
| Residuals (within columns) | 38 | 386.96 | 10.183 |
| Total | 41 | 1348.5 | |

F = 31.474 =(MStreatment/MSresidual)

Summary of Data

| **Group** | **Number of Points** | **Mean** | **Standard Deviation** | **Standard Error of Mean** | **Median** |
|---|---|---|---|---|---|
| GFP | 9 | 30.146 | 1.220 | 0.4066 | 30.631 |
| Xotx5b | 10 | 38.318 | 2.331 | 0.7371 | 37.818 |
| Xotx2 | 9 | 25.976 | 1.790 | 0.5965 | 26.786 |
| Xotx2Mut3/1 | 14 | 36.434 | 4.808 | 1.285 | 36.420 |

| **Group** | **Minimum** | **Maximum** | **95% Confidence Interval From** | **To** |
|---|---|---|---|---|
| GFP | 27.143 | 31.250 | 29.208 | 31.084 |
| Xotx5b | 36.364 | 44.262 | 36.650 | 39.985 |
| Xotx2 | 22.034 | 27.660 | 24.601 | 27.352 |
| Xotx2Mut3/1 | 30.667 | 44.262 | 33.658 | 39.209 |

* * *

Ganglion cells

## One-way Analysis of Variance (ANOVA)

The P value is 0.0241, considered significant.
Variation among column means is significantly greater than expected by chance.

## Tukey-Kramer Multiple Comparisons Test

If the value of q is greater than 3.802 then the P value is less than 0.05.

| **Comparison** | **Mean Difference** | **q** | **P value** | |
|---|---|---|---|---|
| GFP vs Xotx5b | 3.576 | 3.222 | ns | P>0.05 |
| GFP vs Xotx2 | 0.5766 | 0.5064 | ns | P>0.05 |
| GFP vs Xotx2Mut3/1 | 3.824 | 3.706 | ns | P>0.05 |
| Xotx5b vs Xotx2 | -2.999 | 2.703 | ns | P>0.05 |
| Xotx5b vs Xotx2Mut3/1 | 0.2483 | 0.2483 | ns | P>0.05 |
| Xotx2 vs Xotx2Mut3/1 | 3.248 | 3.147 | ns | P>0.05 |

| **Difference** | **Mean Difference** | **95% Confidence Interval From** | **To** |
|---|---|---|---|
| GFP - Xotx5b | 3.576 | -0.6430 | 7.795 |
| GFP - Xotx2 | 0.5766 | -3.752 | 4.905 |
| GFP - Xotx2Mut3/1 | 3.824 | -0.09885 | 7.747 |
| Xotx5b - Xotx2 | -2.999 | -7.218 | 1.220 |
| Xotx5b - Xotx2Mut3/1 | 0.2483 | -3.554 | 4.050 |
| Xotx2 - Xotx2Mut3/1 | 3.248 | -0.6754 | 7.171 |

## Assumption test: Are the standard deviations of the groups equal?

ANOVA assumes that the data are sampled from populations with identical SDs. This assumption is tested using the method of Bartlett.

Bartlett statistic (corrected) = 3.523
The P value is 0.3178.
Bartlett's test suggests that the differences among the SDs is not significant.

## Assumption test: Are the data sampled from Gaussian distributions?

ANOVA assumes that the data are sampled from populations that follow Gaussian distributions. This assumption is tested using the method Kolmogorov and Smirnov:

| **Group** | **KS** | **P Value** | **Passed normality test?** |
|---|---|---|---|
| GFP | 0.1481 | >0.10 | Yes |
| Xotx5b | 0.1987 | >0.10 | Yes |
| Xotx2 | 0.2671 | >0.10 | Yes |

| | | |
|---|---|---|
| Xotx2Mut3/1 | 0.1486 >0.10 | Yes |

Intermediate calculations. ANOVA table

| Source of variation | Degrees of freedom | Sum of squares | Mean square |
|---|---|---|---|
| Treatments (between columns) | 3 | 123.04 | 41.013 |
| Residuals (within columns) | 38 | 443.33 | 11.666 |
| Total | 41 | 566.37 | |

F = 3.515 =(MStreatment/MSresidual)

Summary of Data

| **Group** | **Number of Points** | **Mean** | **Standard Deviation** | **Standard Error of Mean** | **Median** |
|---|---|---|---|---|---|
| GFP | 9 | 17.667 | 2.058 | 0.6861 | 17.308 |
| Xotx5b | 10 | 14.091 | 4.016 | 1.270 | 13.076 |
| Xotx2 | 9 | 17.091 | 3.748 | 1.249 | 15.000 |
| Xotx2Mut3/1 | 14 | 13.843 | 3.418 | 0.9136 | 13.398 |

| | | | **95% Confidence Interval** | |
|---|---|---|---|---|
| **Group** | **Minimum** | **Maximum** | **From** | **To** |
| GFP | 14.583 | 20.896 | 16.085 | 19.249 |
| Xotx5b | 9.836 | 21.212 | 11.218 | 16.964 |
| Xotx2 | 13.077 | 22.034 | 14.210 | 19.972 |
| Xotx2Mut3/1 | 8.333 | 19.565 | 11.870 | 15.816 |

*   *   *

Horizontal cells

## One-way Analysis of Variance (ANOVA)

The P value is 0.0230, considered significant.
Variation among column means is significantly greater than expected by chance.

### Tukey-Kramer Multiple Comparisons Test

If the value of q is greater than 3.802 then the P value is less than 0.05.

| Comparison | Mean Difference | q | | P value |
|---|---|---|---|---|
| GFP vs Xotx5b | 2.773 | 3.676 | ns | P>0.05 |
| GFP vs Xotx2 | 2.306 | 2.980 | ns | P>0.05 |
| GFP vs Xotx2Mut3/1 | 0.3919 | 0.5588 | ns | P>0.05 |
| Xotx5b vs Xotx2 | -0.4663 | 0.6183 | ns | P>0.05 |
| Xotx5b vs Xotx2Mut3/1 | -2.381 | 3.503 | ns | P>0.05 |
| Xotx2 vs Xotx2Mut3/1 | -1.914 | 2.730 | ns | P>0.05 |

| Difference | Mean Difference | 95% Confidence Interval From | To |
|---|---|---|---|
| GFP - Xotx5b | 2.773 | -0.09475 | 5.640 |
| GFP - Xotx2 | 2.306 | -0.6356 | 5.248 |
| GFP - Xotx2Mut3/1 | 0.3919 | -2.274 | 3.058 |
| Xotx5b - Xotx2 | -0.4663 | -3.334 | 2.401 |
| Xotx5b - Xotx2Mut3/1 | -2.381 | -4.965 | 0.2031 |
| Xotx2 - Xotx2Mut3/1 | -1.914 | -4.581 | 0.7519 |

### Assumption test: Are the standard deviations of the groups equal?

ANOVA assumes that the data are sampled from populations with identical SDs. This assumption is tested using the method of Bartlett.

Bartlett statistic (corrected) = 0.4805
The P value is 0.9232.
Bartlett's test suggests that the differences among the SDs is not significant.

### Assumption test: Are the data sampled from Gaussian distributions?

ANOVA assumes that the data are sampled from populations that follow Gaussian distributions. This assumption is tested using the method Kolmogorov and Smirnov:

| Group | KS | P Value | Passed normality test? |
|---|---|---|---|
| GFP | 0.2394 | >0.10 | Yes |
| Xotx5b | 0.1263 | >0.10 | Yes |
| Xotx2 | 0.2466 | >0.10 | Yes |

| | | |
|---|---|---|
| Xotx2Mut3/1 | 0.1470 >0.10 | Yes |

Intermediate calculations. ANOVA table

| Source of variation | Degrees of freedom | Sum of squares | Mean square |
|---|---|---|---|
| Treatments (between columns) | 3 | 57.543 | 19.181 |
| Residuals (within columns) | 38 | 204.77 | 5.389 |
| Total | 41 | 262.32 | |

F = 3.559 =(MStreatment/MSresidual)

Summary of Data

| **Group** | **Number of Points** | **Mean** | **Standard Deviation** | **Standard Error of Mean** | **Median** |
|---|---|---|---|---|---|
| GFP | 9 | 7.481 | 2.196 | 0.7321 | 6.771 |
| Xotx5b | 10 | 4.708 | 2.633 | 0.8328 | 5.137 |
| Xotx2 | 9 | 5.174 | 2.334 | 0.7780 | 5.085 |
| Xotx2Mut3/1 | 14 | 7.089 | 2.152 | 0.5751 | 7.065 |

| **Group** | **Minimum** | **Maximum** | **95% Confidence Interval From** | **To** |
|---|---|---|---|---|
| GFP | 5.405 | 11.429 | 5.793 | 9.169 |
| Xotx5b | 0.000 | 8.197 | 2.824 | 6.592 |
| Xotx2 | 1.923 | 9.231 | 3.380 | 6.969 |
| Xotx2Mut3/1 | 3.125 | 9.836 | 5.847 | 8.331 |

* * *
