# Supplementary material for: A specific box switches the cell fate determining activity of XOTX2 and XOTX5b in the Xenopus retina
Source: Neural Dev. 2007 Jun 27;2:12. doi: 10.1186/1749-8104-2-12 (PMC1929070; doi:10.1186/1749-8104-2-12)
Supplement: Additional file 7 — Results of the statistical ANOVA analysis on neuronal cell type frequency in lipofection experiments with the Xotx deletion constructs. Results of the statistical ANOVA analysis on neuronal cell type frequency in lipofection experiments with the Xotx deletion constructs (Figure 3f). [file 1749-8104-2-12-S7.pdf]

Amacrine cells

One-way Analysis of Variance (ANOVA)

The P value is 0.0172, considered significant.
Variation among column means is significantly greater than expected by chance.

Tukey-Kramer Multiple Comparisons Test

If the value of q is greater than 4.064 then the P value is less than 0.05.

Comparison	Mean Difference	q		P value
GFP vs Xotx5b	-0.6875	1.193	ns	P>0.05
GFP vs Xotx2	1.872	2.878	ns	P>0.05
GFP vs Xotx5bDII	1.323	2.135	ns	P>0.05
GFP vs Xotx2DII	-1.048	1.759	ns	P>0.05
Xotx5b vs Xotx2	2.559	3.789	ns	P>0.05
Xotx5b vs Xotx5bDII	2.011	3.113	ns	P>0.05
Xotx5b vs Xotx2DII	-0.3604	0.5786	ns	P>0.05
Xotx2 vs Xotx5bDII	-0.5487	0.7695	ns	P>0.05
Xotx2 vs Xotx2DII	-2.920	4.218	*	P<0.05
Xotx5bDII vs Xotx2DII	-2.371	3.574	ns	P>0.05

Difference	Mean Difference	95% Confidence Interval From	To
GFP - Xotx5b	-0.6875	-3.029	1.654
GFP - Xotx2	1.872	-0.7719	4.516
GFP - Xotx5bDII	1.323	-1.195	3.842
GFP - Xotx2DII	-1.048	-3.468	1.373
Xotx5b - Xotx2	2.559	-0.1861	5.305
Xotx5b - Xotx5bDII	2.011	-0.6146	4.636
Xotx5b - Xotx2DII	-0.3604	-2.892	2.171
Xotx2 - Xotx5bDII	-0.5487	-3.447	2.349
Xotx2 - Xotx2DII	-2.920	-5.733	-0.1064
Xotx5bDII - Xotx2DII	-2.371	-5.067	0.3251

Assumption test: Are the standard deviations of the groups equal?

ANOVA assumes that the data are sampled from populations with identical SDs. This assumption is tested using the method of Bartlett.

Bartlett statistic (corrected) = 18.661
The P value is 0.0009.
Bartlett's test suggests that the differences among the SDs is extremely significant.
Since ANOVA assumes populations with equal SDs, you should consider transforming your data (reciprocal or log) or selecting a nonparametric test.

Assumption test: Are the data sampled from Gaussian distributions?

ANOVA assumes that the data are sampled from populations that follow Gaussian distributions. This assumption is tested using the method Kolmogorov and Smirnov:

**Group**	**KS**	**P Value**	**Passed normality test?**
GFP	0.1353	>0.10	Yes
Xotx5b	0.2177	>0.10	Yes
Xotx2	0.2831	>0.10	Yes
Xotx5bDII	0.1563	>0.10	Yes
Xotx2DII	0.2063	>0.10	Yes

Intermediate calculations. ANOVA table

Source of variation	Degrees of freedom	Sum of squares	Mean square
Treatments (between columns)	4	45.461	11.365
Residuals (within columns)	36	118.29	3.286
Total	40	163.75	

F = 3.459 =(MStreatment/MSresidual)

Summary of Data

**Group**	**Number of Points**	**Mean**	**Standard Deviation**	**Standard Error of Mean**	**Median**
GFP	11	13.928	1.649	0.4971	13.725
Xotx5b	9	14.615	1.623	0.5410	15.294
Xotx2	6	12.056	3.521	1.437	11.605
Xotx5bDII	7	12.605	0.9401	0.3553	12.403
Xotx2DII	8	14.976	0.6274	0.2218	14.979

			**95% Confidence Interval**	
**Group**	**Minimum**	**Maximum**	**From**	**To**
GFP	11.348	17.323	12.820	15.035
Xotx5b	11.765	16.552	13.368	15.863
Xotx2	8.333	18.519	8.360	15.751
Xotx5bDII	11.203	13.953	11.735	13.474
Xotx2DII	14.338	16.272	14.451	15.500

* * *

Bipolar cells

One-way Analysis of Variance (ANOVA)

The P value is < 0.0001, considered extremely significant.
Variation among column means is significantly greater than expected by chance.

Tukey-Kramer Multiple Comparisons Test

If the value of q is greater than 4.064 then the P value is less than 0.05.

Comparison	Mean Difference	q		P value
GFP vs Xotx5b	3.848	3.643	ns	P>0.05
GFP vs Xotx2	-6.558	5.497	**	P<0.01
GFP vs Xotx5bDII	-0.6893	0.6066	ns	P>0.05
GFP vs Xotx2DII	0.8468	0.7753	ns	P>0.05
Xotx5b vs Xotx2	-10.406	8.400	***	P<0.001
Xotx5b vs Xotx5bDII	-4.538	3.831	ns	P>0.05
Xotx5b vs Xotx2DII	-3.002	2.628	ns	P>0.05
Xotx2 vs Xotx5bDII	5.868	4.488	*	P<0.05
Xotx2 vs Xotx2DII	7.404	5.833	**	P<0.01
Xotx5bDII vs Xotx2DII	1.536	1.263	ns	P>0.05

Difference	Mean Difference	95% Confidence Interval From	To
GFP - Xotx5b	3.848	-0.4453	8.142
GFP - Xotx2	-6.558	-11.406	-1.709
GFP - Xotx5bDII	-0.6893	-5.308	3.929
GFP - Xotx2DII	0.8468	-3.592	5.286
Xotx5b - Xotx2	-10.406	-15.441	-5.371
Xotx5b - Xotx5bDII	-4.538	-9.352	0.2765
Xotx5b - Xotx2DII	-3.002	-7.643	1.640
Xotx2 - Xotx5bDII	5.868	0.5536	11.183
Xotx2 - Xotx2DII	7.404	2.245	12.564
Xotx5bDII - Xotx2DII	1.536	-3.408	6.480

Assumption test: Are the standard deviations of the groups equal?

ANOVA assumes that the data are sampled from populations with identical SDs. This assumption is tested using the method of Bartlett.

Bartlett statistic (corrected) = 6.877
The P value is 0.1425.
Bartlett's test suggests that the differences among the SDs is not significant.

Assumption test: Are the data sampled from Gaussian distributions?

ANOVA assumes that the data are sampled from populations that follow

Gaussian distributions. This assumption is tested using the method Kolmogorov and Smirnov:

Group	KS	P Value	Passed normality test?
GFP	0.1908	>0.10	Yes
Xotx5b	0.2895	>0.10	Yes
Xotx2	0.1641	>0.10	Yes
Xotx5bDII	0.2325	>0.10	Yes
Xotx2DII	0.1763	>0.10	Yes

Intermediate calculations. ANOVA table

Source of variation	Degrees of freedom	Sum of squares	Mean square
Treatments (between columns)	4	400.19	100.05
Residuals (within columns)	36	397.78	11.050
Total	40	797.97	

F = 9.054 =(MStreatment/MSresidual)

Summary of Data

Group	Number of Points	Mean	Standard Deviation	Standard Error of Mean	Median
GFP	11	32.680	4.260	1.284	31.915
Xotx5b	9	28.831	2.002	0.6674	27.861
Xotx2	6	39.238	2.733	1.116	39.535
Xotx5bDII	7	33.369	4.286	1.620	35.150
Xotx2DII	8	31.833	2.288	0.8091	31.626

Group	Minimum	Maximum	95% Confidence Interval From	To
GFP	23.214	38.780	29.818	35.541
Xotx5b	27.273	33.103	27.292	30.371
Xotx2	35.227	42.424	36.368	42.107
Xotx5bDII	26.585	38.365	29.405	37.333
Xotx2DII	29.044	35.470	29.920	33.746

* * *

Photoreceptors

One-way Analysis of Variance (ANOVA)

The P value is < 0.0001, considered extremely significant.
Variation among column means is significantly greater than expected by chance.

Tukey-Kramer Multiple Comparisons Test

If the value of q is greater than 4.064 then the P value is less than 0.05.

Comparison	Mean Difference	q		P value
GFP vs Xotx5b	-7.190	10.541	***	P<0.001
GFP vs Xotx2	3.824	4.964	*	P<0.05
GFP vs Xotx5bDII	0.3378	0.4604	ns	P>0.05
GFP vs Xotx2DII	-0.8220	1.166	ns	P>0.05
Xotx5b vs Xotx2	11.014	13.770	***	P<0.001
Xotx5b vs Xotx5bDII	7.528	9.843	***	P<0.001
Xotx5b vs Xotx2DII	6.368	8.636	***	P<0.001
Xotx2 vs Xotx5bDII	-3.486	4.129	*	P<0.05
Xotx2 vs Xotx2DII	-4.646	5.668	**	P<0.01
Xotx5bDII vs Xotx2DII	-1.160	1.477	ns	P>0.05

Difference	Mean Difference	95% Confidence Interval From	To
GFP - Xotx5b	-7.190	-9.962	-4.418
GFP - Xotx2	3.824	0.6933	6.954
GFP - Xotx5bDII	0.3378	-2.644	3.320
GFP - Xotx2DII	-0.8220	-3.688	2.044
Xotx5b - Xotx2	11.014	7.763	14.264
Xotx5b - Xotx5bDII	7.528	4.420	10.636
Xotx5b - Xotx2DII	6.368	3.371	9.365
Xotx2 - Xotx5bDII	-3.486	-6.917	-0.05435
Xotx2 - Xotx2DII	-4.646	-7.976	-1.315
Xotx5bDII - Xotx2DII	-1.160	-4.352	2.032

Assumption test: Are the standard deviations of the groups equal?

ANOVA assumes that the data are sampled from populations with identical SDs. This assumption is tested using the method of Bartlett.

Bartlett statistic (corrected) = 2.662
The P value is 0.6159.
Bartlett's test suggests that the differences among the SDs is not significant.

Assumption test: Are the data sampled from Gaussian distributions?

ANOVA assumes that the data are sampled from populations that follow

Gaussian distributions. This assumption is tested using the method Kolmogorov and Smirnov:

Group	KS	P Value	Passed normality test?
GFP	0.1897	>0.10	Yes
Xotx5b	0.1738	>0.10	Yes
Xotx2	0.2923	>0.10	Yes
Xotx5bDII	0.2100	>0.10	Yes
Xotx2DII	0.1308	>0.10	Yes

Intermediate calculations. ANOVA table

Source of variation	Degrees of freedom	Sum of squares	Mean square
Treatments (between columns)	4	507.65	126.91
Residuals (within columns)	36	165.82	4.606
Total	40	673.47	

F = 27.554 =(MStreatment/MSresidual)

Summary of Data

Group	Number of Points	Mean	Standard Deviation	Standard Error of Mean	Median
GFP	11	28.146	2.747	0.8283	27.386
Xotx5b	9	35.337	1.807	0.6024	34.826
Xotx2	6	24.323	2.114	0.8629	25.026
Xotx5bDII	7	27.809	1.616	0.6108	27.444
Xotx2DII	8	28.968	1.935	0.6843	29.173

Group	Minimum	Maximum	95% Confidence Interval From	To
GFP	25.490	33.598	26.301	29.992
Xotx5b	33.103	39.216	33.948	36.726
Xotx2	21.212	26.316	22.104	26.541
Xotx5bDII	25.581	30.879	26.314	29.303
Xotx2DII	26.471	32.374	27.350	30.587

* * *

Ganglion cells

One-way Analysis of Variance (ANOVA)

The P value is 0.0087, considered very significant.
Variation among column means is significantly greater than expected by chance.

Tukey-Kramer Multiple Comparisons Test

If the value of q is greater than 4.064 then the P value is less than 0.05.

Comparison	Mean Difference	q		P value
GFP vs Xotx5b	3.833	4.469	*	P<0.05
GFP vs Xotx2	0.5571	0.5753	ns	P>0.05
GFP vs Xotx5bDII	-0.9476	1.027	ns	P>0.05
GFP vs Xotx2DII	1.882	2.123	ns	P>0.05
Xotx5b vs Xotx2	-3.276	3.257	ns	P>0.05
Xotx5b vs Xotx5bDII	-4.780	4.971	*	P<0.05
Xotx5b vs Xotx2DII	-1.950	2.103	ns	P>0.05
Xotx2 vs Xotx5bDII	-1.505	1.417	ns	P>0.05
Xotx2 vs Xotx2DII	1.325	1.286	ns	P>0.05
Xotx5bDII vs Xotx2DII	2.830	2.865	ns	P>0.05

Difference	Mean Difference	95% Confidence Interval From	To
GFP - Xotx5b	3.833	0.3468	7.319
GFP - Xotx2	0.5571	-3.379	4.493
GFP - Xotx5bDII	-0.9476	-4.697	2.802
GFP - Xotx2DII	1.882	-1.721	5.486
Xotx5b - Xotx2	-3.276	-7.363	0.8120
Xotx5b - Xotx5bDII	-4.780	-8.689	-0.8719
Xotx5b - Xotx2DII	-1.950	-5.719	1.818
Xotx2 - Xotx5bDII	-1.505	-5.820	2.810
Xotx2 - Xotx2DII	1.325	-2.863	5.514
Xotx5bDII - Xotx2DII	2.830	-1.184	6.844

Assumption test: Are the standard deviations of the groups equal?

ANOVA assumes that the data are sampled from populations with identical SDs. This assumption is tested using the method of Bartlett.

Bartlett statistic (corrected) = 10.056
The P value is 0.0395.
Bartlett's test suggests that the differences among the SDs is significant.
Since ANOVA assumes populations with equal SDs, you should consider transforming your data (reciprocal or log) or selecting a nonparametric test.

Assumption test: Are the data sampled from Gaussian distributions?

ANOVA assumes that the data are sampled from populations that follow Gaussian distributions. This assumption is tested using the method Kolmogorov and Smirnov:

**Group**	**KS**	**P Value**	**Passed normality test?**
GFP	0.1602	>0.10	Yes
Xotx5b	0.2294	>0.10	Yes
Xotx2	0.2830	>0.10	Yes
Xotx5bDII	0.2613	>0.10	Yes
Xotx2DII	0.2126	>0.10	Yes

Intermediate calculations. ANOVA table

Source of variation	Degrees of freedom	Sum of squares	Mean square
Treatments (between columns)	4	116.50	29.126
Residuals (within columns)	36	262.20	7.283
Total	40	378.70	

F = 3.999 =(MStreatment/MSresidual)

Summary of Data

**Group**	**Number of Points**	**Mean**	**Standard Deviation**	**Standard Error of Mean**	**Median**
GFP	11	20.425	4.009	1.209	20.000
Xotx5b	9	16.592	1.430	0.4767	17.094
Xotx2	6	19.868	1.723	0.7036	19.676
Xotx5bDII	7	21.372	2.222	0.8399	20.930
Xotx2DII	8	18.542	2.410	0.8520	18.695

			**95% Confidence Interval**	
**Group**	**Minimum**	**Maximum**	**From**	**To**
GFP	14.815	26.950	17.732	23.118
Xotx5b	13.793	17.969	15.493	17.691
Xotx2	18.182	21.805	18.059	21.676
Xotx5bDII	18.239	23.643	19.317	23.427
Xotx2DII	14.957	22.426	16.527	20.557

* * *

Horizontal cells

One-way Analysis of Variance (ANOVA)

The P value is 0.4059, considered not significant.
Variation among column means is not significantly greater than expected by chance.

Post tests
Post tests were not calculated because the P value was greater than 0.05.

Assumption test: Are the standard deviations of the groups equal?

ANOVA assumes that the data are sampled from populations with identical SDs. This assumption is tested using the method of Bartlett.

Bartlett statistic (corrected) = 5.784
The P value is 0.2159.
Bartlett's test suggests that the differences among the SDs is not significant.

Assumption test: Are the data sampled from Gaussian distributions?

ANOVA assumes that the data are sampled from populations that follow Gaussian distributions. This assumption is tested using the method Kolmogorov and Smirnov:

**Group**	**KS**	**P Value**	**Passed normality test?**
GFP	0.3156	>0.10	Yes
Xotx5b	0.1504	>0.10	Yes
Xotx2	0.1770	>0.10	Yes
Xotx5bDII	0.2845	>0.10	Yes
Xotx2DII	0.2243	>0.10	Yes

Intermediate calculations. ANOVA table

Source of variation	Degrees of freedom	Sum of squares	Mean square
Treatments (between columns)	4	6.527	1.632
Residuals (within columns)	36	57.121	1.587
Total	40	63.648	

F = 1.028 =(MStreatment/MSresidual)

Summary of Data

**Group**	**Number of Points**	**Mean**	**Standard Deviation**	**Standard Error of Mean**	**Median**
GFP	11	4.822	1.633	0.4923	4.965
Xotx5b	9	4.625	0.8130	0.2710	4.430

Xotx2	6	4.517	1.530	0.6245	4.356
Xotx5bDII	7	4.845	0.8257	0.3121	4.564
Xotx2DII	8	5.681	1.158	0.4092	5.370

			**95% Confidence Interval**	
**Group**	**Minimum**	**Maximum**	**From**	**To**
GFP	2.397	8.929	3.725	5.918
Xotx5b	3.448	6.061	4.000	5.250
Xotx2	2.256	6.366	2.911	6.122
Xotx5bDII	4.070	6.394	4.081	5.609
Xotx2DII	4.250	7.721	4.713	6.649

* * *
