# Supplementary material for: A specific box switches the cell fate determining activity of XOTX2 and XOTX5b in the Xenopus retina
Source: Neural Dev. 2007 Jun 27;2:12. doi: 10.1186/1749-8104-2-12 (PMC1929070; doi:10.1186/1749-8104-2-12)
Supplement: Additional file 8 — Results of the statistical ANOVA analysis on neuronal cell type frequency in lipofection experiments with the otd/Xotx constructs. Results of the statistical ANOVA analysis on neuronal cell type frequency in lipofection experiments with the otd/Xotx constructs (Figure 4b). [file 1749-8104-2-12-S8.pdf]

Amacrine cells

One-way Analysis of Variance (ANOVA)

The P value is 0.0020, considered very significant.
Variation among column means is significantly greater than expected by chance.

Tukey-Kramer Multiple Comparisons Test

If the value of q is greater than 3.974 then the P value is less than 0.05.

**Comparison**	**Mean Difference**	**q**		**P value**
GFP vs Xotx5b	4.408	5.429	**	P<0.01
GFP vs Xotx2	1.738	2.235	ns	P>0.05
GFP vs otd/Xotx2	2.538	2.708	ns	P>0.05
GFP vs otd/Xotx5b	4.143	4.694	*	P<0.05
Xotx5b vs Xotx2	-2.670	3.435	ns	P>0.05
Xotx5b vs otd/Xotx2	-1.869	1.994	ns	P>0.05
Xotx5b vs otd/Xotx5b	-0.2649	0.3001	ns	P>0.05
Xotx2 vs otd/Xotx2	0.8009	0.8823	ns	P>0.05
Xotx2 vs otd/Xotx5b	2.405	2.827	ns	P>0.05
otd/Xotx2 vs otd/Xotx5b	1.604	1.605	ns	P>0.05

**Difference**	**Mean Difference**	**95% Confidence Interval From**	**To**
GFP - Xotx5b	4.408	1.181	7.634
GFP - Xotx2	1.738	-1.351	4.827
GFP - otd/Xotx2	2.538	-1.187	6.264
GFP - otd/Xotx5b	4.143	0.6353	7.650
Xotx5b - Xotx2	-2.670	-5.759	0.4189
Xotx5b - otd/Xotx2	-1.869	-5.595	1.856
Xotx5b - otd/Xotx5b	-0.2649	-3.772	3.243
Xotx2 - otd/Xotx2	0.8009	-2.806	4.408
Xotx2 - otd/Xotx5b	2.405	-0.9763	5.787
otd/Xotx2 - otd/Xotx5b	1.604	-2.367	5.576

Assumption test: Are the standard deviations of the groups equal?

ANOVA assumes that the data are sampled from populations with identical SDs. This assumption is tested using the method of Bartlett.

Bartlett statistic (corrected) = 5.293
The P value is 0.2586.
Bartlett's test suggests that the differences among the SDs is not significant.

Assumption test: Are the data sampled from Gaussian distributions?

ANOVA assumes that the data are sampled from populations that follow

Gaussian distributions. This assumption is tested using the method Kolmogorov and Smirnov:

Group	KS	P Value	Passed normality test?
GFP	0.1229	>0.10	Yes
Xotx5b	0.2231	>0.10	Yes
Xotx2	0.1431	>0.10	Yes
otd/Xotx2	0.2179	>0.10	Yes
otd/Xotx5b	0.1357	>0.10	Yes

Intermediate calculations. ANOVA table

Source of variation	Degrees of freedom	Sum of squares	Mean square
Treatments (between columns)	4	188.24	47.059
Residuals (within columns)	63	622.87	9.887
Total	67	811.11	

F = 4.760 =(MStreatment/MSresidual)

Summary of Data

Group	Number of Points	Mean	Standard Deviation	Standard Error of Mean	Median
GFP	15	16.218	3.790	0.9786	16.102
Xotx5b	15	11.810	3.601	0.9299	12.150
Xotx2	18	14.480	2.553	0.6017	14.538
otd/Xotx2	9	13.679	1.994	0.6646	13.971
otd/Xotx5b	11	12.075	3.124	0.9419	11.620

Group	Minimum	Maximum	95% Confidence Interval From	To
GFP	9.524	22.609	14.118	18.317
Xotx5b	1.961	18.293	9.815	13.805
Xotx2	7.692	20.430	13.210	15.750
otd/Xotx2	10.526	15.909	12.147	15.212
otd/Xotx5b	5.882	17.460	9.976	14.173

* * *

Bipolar cells

One-way Analysis of Variance (ANOVA)

The P value is < 0.0001, considered extremely significant.
Variation among column means is significantly greater than expected by chance.

Tukey-Kramer Multiple Comparisons Test

If the value of q is greater than 3.974 then the P value is less than 0.05.

Comparison	Mean Difference	q	P value
GFP vs Xotx5b	-1.456	1.644	ns P>0.05
GFP vs Xotx2	-9.206	10.857	*** P<0.001
GFP vs otd/Xotx2	-7.871	7.696	*** P<0.001
GFP vs otd/Xotx5b	-0.9381	0.9743	ns P>0.05
Xotx5b vs Xotx2	-7.751	9.140	*** P<0.001
Xotx5b vs otd/Xotx2	-6.415	6.273	*** P<0.001
Xotx5b vs otd/Xotx5b	0.5177	0.5377	ns P>0.05
Xotx2 vs otd/Xotx2	1.335	1.349	ns P>0.05
Xotx2 vs otd/Xotx5b	8.268	8.907	*** P<0.001
otd/Xotx2 vs otd/Xotx5b	6.933	6.359	*** P<0.001

Difference	Mean Difference	95% Confidence Interval From	To
GFP - Xotx5b	-1.456	-4.976	2.064
GFP - Xotx2	-9.206	-12.576	-5.836
GFP - otd/Xotx2	-7.871	-11.935	-3.807
GFP - otd/Xotx5b	-0.9381	-4.764	2.888
Xotx5b - Xotx2	-7.751	-11.121	-4.381
Xotx5b - otd/Xotx2	-6.415	-10.479	-2.351
Xotx5b - otd/Xotx5b	0.5177	-3.309	4.344
Xotx2 - otd/Xotx2	1.335	-2.600	5.271
Xotx2 - otd/Xotx5b	8.268	4.579	11.957
otd/Xotx2 - otd/Xotx5b	6.933	2.600	11.265

Assumption test: Are the standard deviations of the groups equal?

ANOVA assumes that the data are sampled from populations with identical SDs. This assumption is tested using the method of Bartlett.

Bartlett statistic (corrected) = 6.918
The P value is 0.1403.
Bartlett's test suggests that the differences among the SDs is not significant.

Assumption test: Are the data sampled from Gaussian distributions?

ANOVA assumes that the data are sampled from populations that follow

Gaussian distributions. This assumption is tested using the method Kolmogorov and Smirnov:

Group	KS	P Value	Passed normality test?
GFP	0.1836	>0.10	Yes
Xotx5b	0.1675	>0.10	Yes
Xotx2	0.2534	>0.10	Yes
otd/Xotx2	0.2168	>0.10	Yes
otd/Xotx5b	0.1610	>0.10	Yes

Intermediate calculations. ANOVA table

Source of variation	Degrees of freedom	Sum of squares	Mean square
Treatments (between columns)	4	1062.8	265.71
Residuals (within columns)	63	741.32	11.767
Total	67	1804.2	

F = 22.581 =(MStreatment/MSresidual)

Summary of Data

Group	Number of Points	Mean	Standard Deviation	Standard Error of Mean	Median
GFP	15	29.028	2.034	0.5251	28.571
Xotx5b	15	30.484	3.622	0.9352	31.148
Xotx2	18	38.234	3.532	0.8326	38.152
otd/Xotx2	9	36.899	4.515	1.505	36.029
otd/Xotx5b	11	29.966	3.529	1.064	28.929

Group	Minimum	Maximum	95% Confidence Interval From	To
GFP	26.667	33.333	27.901	30.154
Xotx5b	24.735	35.000	28.478	32.490
Xotx2	34.409	50.000	36.477	39.991
otd/Xotx2	32.121	47.368	33.428	40.369
otd/Xotx5b	25.000	35.294	27.595	32.337

* * *

Photoreceptors

One-way Analysis of Variance (ANOVA)

The P value is < 0.0001, considered extremely significant.
Variation among column means is significantly greater than expected by chance.

Tukey-Kramer Multiple Comparisons Test

If the value of q is greater than 3.974 then the P value is less than 0.05.

**Comparison**	**Mean Difference**	**q**		**P value**
GFP vs Xotx5b	-9.406	9.110	***	P<0.001
GFP vs Xotx2	6.430	6.504	***	P<0.001
GFP vs otd/Xotx2	0.02300	0.01929	ns	P>0.05
GFP vs otd/Xotx5b	-4.633	4.127	*	P<0.05
Xotx5b vs Xotx2	15.835	16.019	***	P<0.001
Xotx5b vs otd/Xotx2	9.429	7.908	***	P<0.001
Xotx5b vs otd/Xotx5b	4.773	4.252	*	P<0.05
Xotx2 vs otd/Xotx2	-6.407	5.550	**	P<0.01
Xotx2 vs otd/Xotx5b	-11.062	10.223	***	P<0.001
otd/Xotx2 vs otd/Xotx5b	-4.656	3.663	ns	P>0.05

**Difference**	**Mean Difference**	**95% Confidence Interval From**	**To**
GFP - Xotx5b	-9.406	-13.509	-5.302
GFP - Xotx2	6.430	2.501	10.358
GFP - otd/Xotx2	0.02300	-4.715	4.761
GFP - otd/Xotx5b	-4.633	-9.093	-0.1719
Xotx5b - Xotx2	15.835	11.907	19.764
Xotx5b - otd/Xotx2	9.429	4.691	14.167
Xotx5b - otd/Xotx5b	4.773	0.3125	9.234
Xotx2 - otd/Xotx2	-6.407	-10.994	-1.819
Xotx2 - otd/Xotx5b	-11.062	-15.363	-6.762
otd/Xotx2 - otd/Xotx5b	-4.656	-9.706	0.3951

Assumption test: Are the standard deviations of the groups equal?

ANOVA assumes that the data are sampled from populations with identical SDs. This assumption is tested using the method of Bartlett.

Bartlett statistic (corrected) = 25.821
The P value is < 0.0001.
Bartlett's test suggests that the differences among the SDs is extremely significant.
Since ANOVA assumes populations with equal SDs, you should consider transforming your data (reciprocal or log) or selecting a nonparametric test.

Assumption test: Are the data sampled from Gaussian distributions?

ANOVA assumes that the data are sampled from populations that follow Gaussian distributions. This assumption is tested using the method Kolmogorov and Smirnov:

Group	KS	P Value	Passed normality test?
GFP	0.08800	>0.10	Yes
Xotx5b	0.2178	>0.10	Yes
Xotx2	0.1602	>0.10	Yes
otd/Xotx2	0.1433	>0.10	Yes
otd/Xotx5b	0.1910	>0.10	Yes

Intermediate calculations. ANOVA table

Source of variation	Degrees of freedom	Sum of squares	Mean square
Treatments (between columns)	4	2222.1	555.52
Residuals (within columns)	63	1007.4	15.991
Total	67	3229.5	

F = 34.739 =(MStreatment/MSresidual)

Summary of Data

Group	Number of Points	Mean	Standard Deviation	Standard Error of Mean	Median
GFP	15	30.393	2.861	0.7388	30.000
Xotx5b	15	39.799	5.172	1.335	38.000
Xotx2	18	23.964	2.278	0.5370	23.785
otd/Xotx2	9	30.370	7.001	2.334	31.579
otd/Xotx5b	11	35.026	1.950	0.5878	35.484

Group	Minimum	Maximum	95% Confidence Interval From	95% Confidence Interval To
GFP	26.271	35.766	28.809	31.978
Xotx5b	34.756	51.852	36.935	42.663
Xotx2	19.355	27.404	22.830	25.097
otd/Xotx2	20.455	39.344	24.989	35.752
otd/Xotx5b	31.818	37.870	33.716	36.336

* * *

Ganglion cells

One-way Analysis of Variance (ANOVA)

The P value is 0.0019, considered very significant.
Variation among column means is significantly greater than expected by chance.

Tukey-Kramer Multiple Comparisons Test

If the value of q is greater than 3.974 then the P value is less than 0.05.

Comparison	Mean Difference	q		P value
GFP vs Xotx5b	4.113	4.191	*	P<0.05
GFP vs Xotx2	2.634	2.803	ns	P>0.05
GFP vs otd/Xotx2	4.328	3.819	ns	P>0.05
GFP vs otd/Xotx5b	-0.9305	0.8721	ns	P>0.05
Xotx5b vs Xotx2	-1.479	1.574	ns	P>0.05
Xotx5b vs otd/Xotx2	0.2151	0.1898	ns	P>0.05
Xotx5b vs otd/Xotx5b	-5.044	4.727	*	P<0.05
Xotx2 vs otd/Xotx2	1.694	1.544	ns	P>0.05
Xotx2 vs otd/Xotx5b	-3.564	3.465	ns	P>0.05
otd/Xotx2 vs otd/Xotx5b	-5.259	4.353	*	P<0.05

Difference	Mean Difference	95% Confidence Interval From	To
GFP - Xotx5b	4.113	0.2125	8.014
GFP - Xotx2	2.634	-1.101	6.368
GFP - otd/Xotx2	4.328	-0.1758	8.832
GFP - otd/Xotx5b	-0.9305	-5.171	3.310
Xotx5b - Xotx2	-1.479	-5.214	2.255
Xotx5b - otd/Xotx2	0.2151	-4.289	4.719
Xotx5b - otd/Xotx5b	-5.044	-9.284	-0.8033
Xotx2 - otd/Xotx2	1.694	-2.667	6.055
Xotx2 - otd/Xotx5b	-3.564	-7.653	0.5238
otd/Xotx2 - otd/Xotx5b	-5.259	-10.060	-0.4574

Assumption test: Are the standard deviations of the groups equal?

ANOVA assumes that the data are sampled from populations with identical SDs. This assumption is tested using the method of Bartlett.

Bartlett statistic (corrected) = 4.267
The P value is 0.3711.
Bartlett's test suggests that the differences among the SDs is not significant.

Assumption test: Are the data sampled from Gaussian distributions?

ANOVA assumes that the data are sampled from populations that follow

Gaussian distributions. This assumption is tested using the method Kolmogorov and Smirnov:

Group	KS	P Value	Passed normality test?
GFP	0.2191	>0.10	Yes
Xotx5b	0.1607	>0.10	Yes
Xotx2	0.1468	>0.10	Yes
otd/Xotx2	0.1868	>0.10	Yes
otd/Xotx5b	0.1903	>0.10	Yes

Intermediate calculations. ANOVA table

Source of variation	Degrees of freedom	Sum of squares	Mean square
Treatments (between columns)	4	277.42	69.354
Residuals (within columns)	63	910.42	14.451
Total	67	1187.8	

F = 4.799 =(MStreatment/MSresidual)

Summary of Data

Group	Number of Points	Mean	Standard Deviation	Standard Error of Mean	Median
GFP	15	15.242	3.480	0.8985	14.365
Xotx5b	15	11.129	3.422	0.8837	10.448
Xotx2	18	12.608	4.061	0.9571	12.528
otd/Xotx2	9	10.914	5.249	1.750	10.294
otd/Xotx5b	11	16.173	2.760	0.8322	16.129

Group	Minimum	Maximum	95% Confidence Interval From	To
GFP	11.429	23.810	13.315	17.169
Xotx5b	3.704	16.260	9.233	13.024
Xotx2	6.452	21.739	10.589	14.628
otd/Xotx2	3.509	19.277	6.879	14.948
otd/Xotx5b	12.088	20.710	14.318	18.027

* * *

Horizontal cells

One-way Analysis of Variance (ANOVA)

The P value is 0.0129, considered significant.
Variation among column means is significantly greater than expected by chance.

Tukey-Kramer Multiple Comparisons Test

If the value of q is greater than 3.974 then the P value is less than 0.05.

**Comparison**	**Mean Difference**	**q**	**P value**	
GFP vs Xotx5b	2.341	2.563	ns	P>0.05
GFP vs Xotx2	-1.595	1.824	ns	P>0.05
GFP vs otd/Xotx2	0.9812	0.9304	ns	P>0.05
GFP vs otd/Xotx5b	2.358	2.375	ns	P>0.05
Xotx5b vs Xotx2	-3.936	4.501	*	P<0.05
Xotx5b vs otd/Xotx2	-1.359	1.289	ns	P>0.05
Xotx5b vs otd/Xotx5b	0.01755	0.01768	ns	P>0.05
Xotx2 vs otd/Xotx2	2.576	2.523	ns	P>0.05
Xotx2 vs otd/Xotx5b	3.953	4.130	*	P<0.05
otd/Xotx2 vs otd/Xotx5b	1.377	1.225	ns	P>0.05

**Difference**	**Mean Difference**	**95% Confidence Interval From**	**To**
GFP - Xotx5b	2.341	-1.289	5.970
GFP - Xotx2	-1.595	-5.070	1.880
GFP - otd/Xotx2	0.9812	-3.210	5.172
GFP - otd/Xotx5b	2.358	-1.588	6.304
Xotx5b - Xotx2	-3.936	-7.410	-0.4606
Xotx5b - otd/Xotx2	-1.359	-5.550	2.832
Xotx5b - otd/Xotx5b	0.01755	-3.928	3.963
Xotx2 - otd/Xotx2	2.576	-1.482	6.634
Xotx2 - otd/Xotx5b	3.953	0.1492	7.757
otd/Xotx2 - otd/Xotx5b	1.377	-3.091	5.844

Assumption test: Are the standard deviations of the groups equal?

ANOVA assumes that the data are sampled from populations with identical SDs. This assumption is tested using the method of Bartlett.

Bartlett statistic (corrected) = 10.120
The P value is 0.0385.
Bartlett's test suggests that the differences among the SDs is significant.
Since ANOVA assumes populations with equal SDs, you should consider transforming your data (reciprocal or log) or selecting a nonparametric test.

Assumption test: Are the data sampled from Gaussian distributions?

ANOVA assumes that the data are sampled from populations that follow Gaussian distributions. This assumption is tested using the method Kolmogorov and Smirnov:

**Group**	**KS**	**P Value**	**Passed normality test?**
GFP	0.1247	>0.10	Yes
Xotx5b	0.1306	>0.10	Yes
Xotx2	0.2069	>0.10	Yes
otd/Xotx2	0.1482	>0.10	Yes
otd/Xotx5b	0.1615	>0.10	Yes

Intermediate calculations. ANOVA table

Source of variation	Degrees of freedom	Sum of squares	Mean square
Treatments (between columns)	4	172.87	43.218
Residuals (within columns)	63	788.22	12.511
Total	67	961.10	

F = 3.454 =(MStreatment/MSresidual)

Summary of Data

**Group**	**Number of Points**	**Mean**	**Standard Deviation**	**Standard Error of Mean**	**Median**
GFP	15	9.119	3.194	0.8247	8.571
Xotx5b	15	6.779	2.891	0.7464	6.504
Xotx2	18	10.714	4.979	1.173	10.148
otd/Xotx2	9	8.138	2.631	0.8769	8.560
otd/Xotx5b	11	6.761	2.273	0.6853	6.690

			**95% Confidence Interval**	
**Group**	**Minimum**	**Maximum**	**From**	**To**
GFP	4.762	15.254	7.350	10.888
Xotx5b	1.724	12.500	5.178	8.380
Xotx2	3.158	25.806	8.238	13.190
otd/Xotx2	4.412	12.821	6.116	10.160
otd/Xotx5b	3.297	10.345	5.234	8.288

* * *
