# Supplementary material for: A specific box switches the cell fate determining activity of XOTX2 and XOTX5b in the Xenopus retina
Source: Neural Dev. 2007 Jun 27;2:12. doi: 10.1186/1749-8104-2-12 (PMC1929070; doi:10.1186/1749-8104-2-12)
Supplement: Additional file 10 — Results of the statistical ANOVA analysis on neuronal cell type frequency in lipofection experiments with the otd/box2 and otd/box5b constructs. Results of the statistical ANOVA analysis on neuronal cell type frequency in lipofection experiments with the otd/box2 and otd/box5b constructs (Figure 4c). [file 1749-8104-2-12-S10.pdf]

amacrine cells

One-way Analysis of Variance (ANOVA)

The P value is 0.4142, considered not significant.
Variation among column means is not significantly greater than expected by chance.

Post tests

Post tests were not calculated because the P value was greater than 0.05.

Assumption test: Are the standard deviations of the groups equal?

ANOVA assumes that the data are sampled from populations with identical SDs. This assumption is tested using the method of Bartlett.

Bartlett statistic (corrected) = 3.957
The P value is 0.1383.
Bartlett's test suggests that the differences among the SDs is not significant.

Assumption test: Are the data sampled from Gaussian distributions?

ANOVA assumes that the data are sampled from populations that follow Gaussian distributions. This assumption is tested using the method Kolmogorov and Smirnov:

**Group**	**KS**	**P Value**	**Passed normality test?**
GFP	0.1411	>0.10	Yes
otd box5b	0.2288	>0.10	Yes
otd box2	0.2530	>0.10	Yes

Intermediate calculations. ANOVA table

Source of variation	Degrees of freedom	Sum of squares	Mean square
Treatments (between columns)	2	16.165	8.083
Residuals (within columns)	21	184.63	8.792
Total	23	200.80	

F = 0.9193 =(MStreatment/MSresidual)

Summary of Data

**Group**	**Number of Points**	**Mean**	**Standard Deviation**	**Standard Error of Mean**	**Median**
GFP	6	14.035	1.398	0.5707	14.063
otd box5b	9	14.926	3.547	1.182	16.312
otd box2	9	16.108	3.046	1.015	17.164

Group	Minimum	Maximum	95% Confidence Interval From	To
GFP	11.856	15.652	12.568	15.503
otd box5b	6.780	18.852	12.200	17.653
otd box2	10.127	20.000	13.767	18.449

* * *

bipolar cells

One-way Analysis of Variance (ANOVA)

The P value is < 0.0001, considered extremely significant.
Variation among column means is significantly greater than expected by chance.

Tukey-Kramer Multiple Comparisons Test

If the value of q is greater than 3.567 then the P value is less than 0.05.

Comparison	Mean Difference	q	P value
GFP vs otd box5b	4.169	3.318	ns P>0.05
GFP vs otd box2	-6.652	5.294	** P<0.01
otd box5b vs otd box2	-10.820	9.629	*** P<0.001

Difference	Mean Difference	95% Confidence Interval From	To
GFP - otd box5b	4.169	-0.3122	8.649
GFP - otd box2	-6.652	-11.132	-2.171
otd box5b - otd box2	-10.820	-14.828	-6.812

Assumption test: Are the standard deviations of the groups equal?

ANOVA assumes that the data are sampled from populations with identical SDs. This assumption is tested using the method of Bartlett.

Bartlett statistic (corrected) = 0.9578
The P value is 0.6195.
Bartlett's test suggests that the differences among the SDs is not significant.

Assumption test: Are the data sampled from Gaussian distributions?

ANOVA assumes that the data are sampled from populations that follow Gaussian distributions. This assumption is tested using the method Kolmogorov and Smirnov:

Group	KS	P Value	Passed normality test?
GFP	0.1276	>0.10	Yes
otd box5b	0.2065	>0.10	Yes
otd box2	0.1533	>0.10	Yes

Intermediate calculations. ANOVA table

Source of variation	Degrees of freedom	Sum of squares	Mean square

Treatments (between columns)	2	533.78	266.89
Residuals (within columns)	21	238.66	11.365
Total	23	772.44	

F = 23.484 =(MStreatment/MSresidual)

Summary of Data

Group	Number of Points	Mean	Standard Deviation	Standard Error of Mean	Median
GFP	6	32.685	3.650	1.490	32.302
otd box5b	9	28.516	3.784	1.261	27.049
otd box2	9	39.336	2.681	0.8937	40.000

Group	Minimum	Maximum	95% Confidence Interval From	95% Confidence Interval To
GFP	28.261	38.415	28.853	36.516
otd box5b	24.528	36.441	25.608	31.425
otd box2	35.753	43.284	37.276	41.397

* * *

photoreceptors

One-way Analysis of Variance (ANOVA)

The P value is < 0.0001, considered extremely significant.
Variation among column means is significantly greater than expected by chance.

Tukey-Kramer Multiple Comparisons Test

If the value of q is greater than 3.567 then the P value is less than 0.05.

Comparison	Mean Difference	q	P value
GFP vs otd box5b	-5.610	5.449	** P<0.01
GFP vs otd box2	1.703	1.654	ns P>0.05
otd box5b vs otd box2	7.313	7.941	*** P<0.001

Difference	Mean Difference	95% Confidence Interval From	To
GFP - otd box5b	-5.610	-9.282	-1.938
GFP - otd box2	1.703	-1.969	5.375
otd box5b - otd box2	7.313	4.029	10.597

Assumption test: Are the standard deviations of the groups equal?

ANOVA assumes that the data are sampled from populations with identical SDs. This assumption is tested using the method of Bartlett.

Bartlett statistic (corrected) = 2.409
The P value is 0.2999.
Bartlett's test suggests that the differences among the SDs is not significant.

Assumption test: Are the data sampled from Gaussian distributions?

ANOVA assumes that the data are sampled from populations that follow Gaussian distributions. This assumption is tested using the method Kolmogorov and Smirnov:

Group	KS	P Value	Passed normality test?
GFP	0.3747	>0.10	Yes
otd box5b	0.2457	>0.10	Yes
otd box2	0.1799	>0.10	Yes

Intermediate calculations. ANOVA table

Source of variation	Degrees of freedom	Sum of squares	Mean square

Treatments (between columns)	2	257.83	128.91
Residuals (within columns)	21	160.27	7.632
Total	23	418.10	

F = 16.891 =(MStreatment/MSresidual)

Summary of Data

Group	Number of Points	Mean	Standard Deviation	Standard Error of Mean	Median
GFP	6	28.056	3.256	1.329	27.053
otd box5b	9	33.666	1.859	0.6197	34.091
otd box2	9	26.353	3.155	1.052	25.714

Group	Minimum	Maximum	95% Confidence Interval From	To
GFP	25.887	34.536	24.638	31.473
otd box5b	29.661	36.066	32.236	35.095
otd box2	23.116	32.258	23.928	28.778

* * *

ganglion cells

One-way Analysis of Variance (ANOVA)

The P value is 0.0015, considered very significant.
Variation among column means is significantly greater than expected by chance.

Tukey-Kramer Multiple Comparisons Test
If the value of q is greater than 3.567 then the P value is less than 0.05.

**Comparison**	**Mean Difference**	**q**		**P value**
GFP vs otd box5b	2.625	2.607	ns	P>0.05
GFP vs otd box2	5.917	5.878	**	P<0.01
otd box5b vs otd box2	3.292	3.656	*	P<0.05

**Difference**	**Mean Difference**	**95% Confidence Interval From**	**To**
GFP - otd box5b	2.625	-0.9654	6.215
GFP - otd box2	5.917	2.327	9.507
otd box5b - otd box2	3.292	0.08095	6.503

Assumption test: Are the standard deviations of the groups equal?

ANOVA assumes that the data are sampled from populations with identical SDs. This assumption is tested using the method of Bartlett.

Bartlett statistic (corrected) = 0.7457
The P value is 0.6888.
Bartlett's test suggests that the differences among the SDs is not significant.

Assumption test: Are the data sampled from Gaussian distributions?

ANOVA assumes that the data are sampled from populations that follow Gaussian distributions. This assumption is tested using the method Kolmogorov and Smirnov:

**Group**	**KS**	**P Value**	**Passed normality test?**
GFP	0.2270	>0.10	Yes
otd box5b	0.3022	>0.10	Yes
otd box2	0.1879	>0.10	Yes

Intermediate calculations. ANOVA table

Source of variation	Degrees of freedom	Sum of squares	Mean square

Treatments (between columns)	2	130.86	65.430
Residuals (within columns)	21	153.23	7.296
Total	23	284.09	

F = 8.967 =(MStreatment/MSresidual)

Summary of Data

Group	Number of Points	Mean	Standard Deviation	Standard Error of Mean	Median
GFP	6	20.614	3.303	1.349	21.305
otd box5b	9	17.989	2.330	0.7765	18.644
otd box2	9	14.697	2.628	0.8760	14.241

Group	Minimum	Maximum	95% Confidence Interval From	To
GFP	16.495	24.457	17.147	24.081
otd box5b	12.295	20.455	16.198	19.780
otd box2	11.429	18.593	12.677	16.717

* * *

horizontal cells

One-way Analysis of Variance (ANOVA)

The P value is 0.2604, considered not significant.
Variation among column means is not significantly greater than expected by chance.

Post tests

Post tests were not calculated because the P value was greater than 0.05.

Assumption test: Are the standard deviations of the groups equal?

ANOVA assumes that the data are sampled from populations with identical SDs. This assumption is tested using the method of Bartlett.

Bartlett statistic (corrected) = 1.734
The P value is 0.4203.
Bartlett's test suggests that the differences among the SDs is not significant.

Assumption test: Are the data sampled from Gaussian distributions?

ANOVA assumes that the data are sampled from populations that follow Gaussian distributions. This assumption is tested using the method Kolmogorov and Smirnov:

Group	KS	P Value	Passed normality test?
GFP	0.2357	>0.10	Yes
otd box5b	0.1683	>0.10	Yes
otd box2	0.2010	>0.10	Yes

Intermediate calculations. ANOVA table

Source of variation	Degrees of freedom	Sum of squares	Mean square
Treatments (between columns)	2	9.523	4.762
Residuals (within columns)	21	69.651	3.317
Total	23	79.174	

F = 1.436 =(MStreatment/MSresidual)

Summary of Data

Group	Number of Points	Mean	Standard Deviation	Standard Error of Mean	Median
GFP	6	4.611	2.036	0.8314	5.016
otd box5b	9	4.903	2.093	0.6978	4.965
otd box2	9	3.506	1.316	0.4388	3.015

Group	Minimum	Maximum	95% Confidence Interval From	To
GFP	1.829	7.216	2.473	6.748
otd box5b	2.198	8.475	3.294	6.512
otd box2	1.613	5.970	2.494	4.518

* * *
